# Assessing the validity of leucine zipper constructs predicted by AlphaFold

**DOI:** 10.1002/pro.70438

**Published:** 2025-12-23

**Authors:** Isobel Mitic, Keiran Rowell, Thomas Litfin, Katharine A. Michie, David A. Jacques

**Affiliations:** ^1^ Department of Molecular Medicine, School of Biomedical Sciences University of New South Wales Sydney New South Wales Australia; ^2^ EMBL Australia Node in Single Molecule Science, School of Biomedical Sciences University of New South Wales Sydney New South Wales Australia; ^3^ Structural Biology Facility, Mark Wainwright Analytical Centre University of New South Wales Sydney New South Wales Australia

**Keywords:** AlphaFold Multimer, AlphaFold2, Boltz‐1, electrostatic interactions, ipTM, leucine zipper, PAE, pLDDT, quality assessment

## Abstract

AP‐1 transcription factors are a network of cellular regulators that combine in different dimer pairs to control a range of pathways involved in differentiation, growth, and cell death. They dimerize via leucine zipper coiled‐coil domains that are preceded by a basic DNA binding domain. Depending on which AP‐1 transcription factors dimerize, different DNA sequences will be recognized resulting in differential gene expression. The affinity of AP‐1 transcription factors for each other dictates which dimers form. The relative concentration of AP‐1 transcription factors varies with tissue type and environment, adding another layer of control to this integral network of cellular regulation. The development of artificial intelligence (AI)‐based protein structure prediction methods gives us a new technique to investigate or predict how dimerization affects combinatorial control. All versions of AlphaFold2 and AlphaFold3 are AI/deep learning programs that predict 3D structures of proteins from an amino acid sequence and multiple sequence alignments of homologous proteins. To fully realize the potential of AI for structural biology, it is essential to understand its current capabilities and limitations. In this study, we used the classical example of an AP‐1 dimer: Fos and Jun, and an array of over 2000 experimentally tested human leucine zippers to interrogate how AlphaFold models leucine zipper domains and if AlphaFold can be used to differentiate between probable and improbable dimer interfaces. We found that AlphaFold predicts highly confident leucine zipper dimers, even for dimer pairs such as the FosB homodimer, for which electrostatics are known to prevent their formation in vivo. This is an important case study concerning high‐confidence but low‐accuracy protein structure prediction.

## 1 INTRODUCTION

**FIGURE 1:**
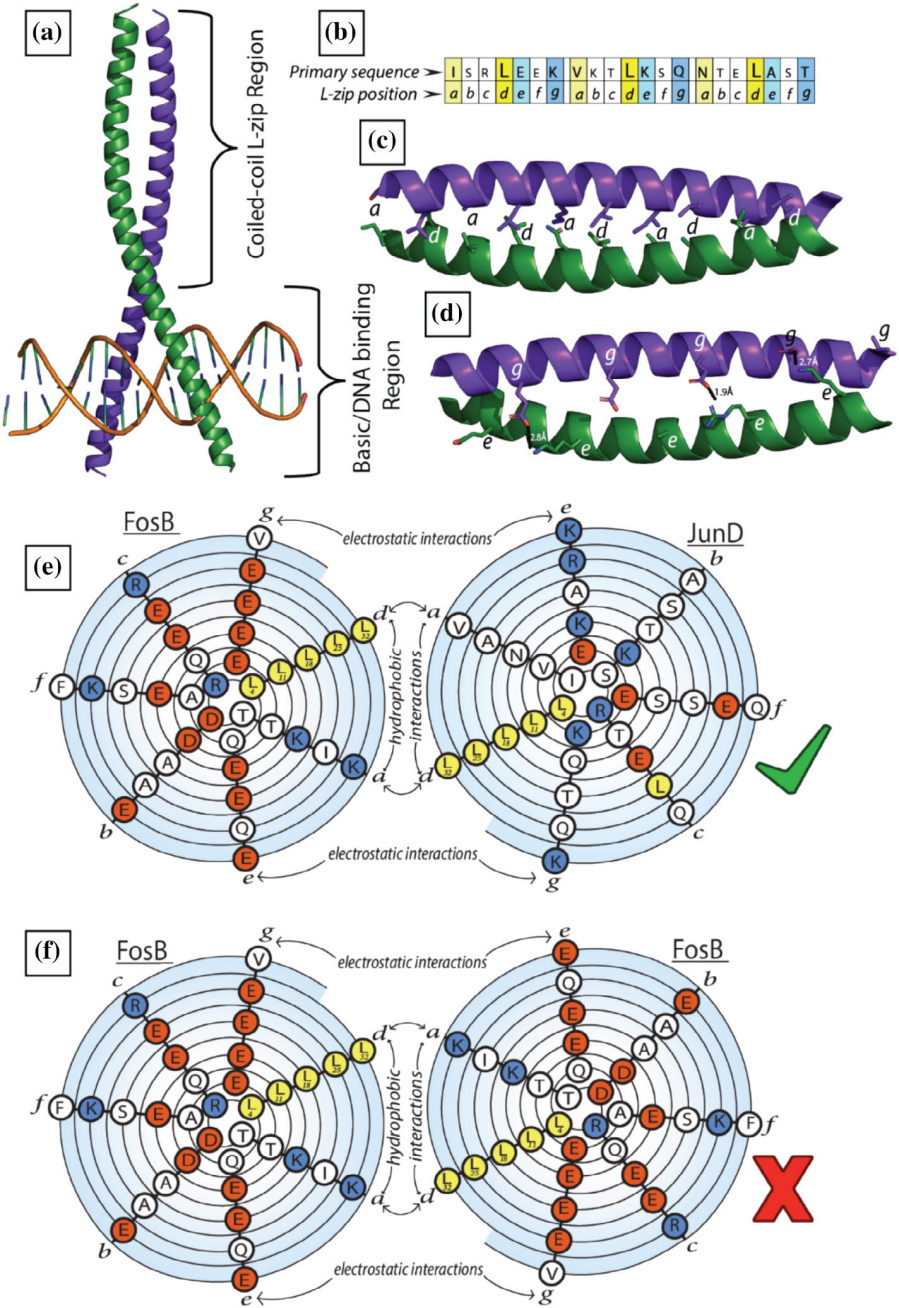
The crystal structure of a Leucine‐zipper. FosB is shown in purple, JunD is shown in green. (a) Crystal structure of FosB and JunD dimer bound to DNA (PDB: 5VPF). (b) Primary sequence of three heptad repeats from JunD, a and d in yellow, e in cyan, g in blue. (c) L‐zip region of FosB‐JunD dimer, a and d residues shown as sticks (PDB: 5VPF). (d) L‐zip region of FosB‐JunD dimer, g residues from FosB and the e residues from JunD shown as sticks (PDB: 5VPF). Interchain bonds shown as dashed black lines. (e) Spiral diagram of the primary sequence of FosB and JunD. The N‐terminus of the L‐zip starts in the center of each spiral, and seven residues are shown over two turns of the spiral diagram. Each spiral is oriented to mimic the structure of the coiled‐coil L‐zip. Leu residues are shown in yellow, negatively charged residues are shown in red and positively charged residues are shown in blue (Branden & Tooze, 1991). (f) Spiral diagram of the primary sequence of FosB in a homodimer.

Fos and Jun were first identified as the oncogenic viral protein vFos and vJun, isolated from avian sarcoma virus, before their eukaryotic homologs were cloned and characterized (Vogt et al., 1987). Since then, Fos and Jun have been incorporated into the larger category of Activator Protein 1 (AP‐1) transcription factors, which are a ubiquitous family of homo or heterodimers with a coiled‐coil Leucine zipper (L‐zip) dimer interface (Bejjani et al., 2019; Yin et al., 2017) (Figure 1a).

AP‐1 transcription factors, including Jun, Fos, Maf, and ATF proteins, regulate a wide range of biological pathways through combinatorial control. These pathways include cell proliferation and apoptotic signaling, classifying AP‐1 transcription factors as both oncogenic and tumor suppressors (Eferl & Wagner, 2003). Furthermore, cell signaling pathways controlled by AP‐1 transcription factors are frequently disrupted or dysregulated by viral and intracellular bacterial infections, contributing to the pathogenicity of these infectious agents (Gazon et al., 2012; Krämer et al., 2015; Zachos et al., 1999). The expression levels of each individual AP‐1 protein vary depending on cell type and environment, and the relative affinity that AP‐1 proteins have for each other varies depending on the primary sequence of their L‐zip coiled‐coil domain. This forms a complex network of transcriptional regulators, based on dimer formation and affinity (Bejjani et al., 2019; Eferl & Wagner, 2003). Perhaps unsurprisingly, the AP‐1 L‐zip dimer interface has been analyzed in atomic detail to understand dimer affinity, specificity and formation (Newman & Keating, 2003; Vinson et al., 2002, 2006).

AP‐1 L‐zips are formed of 4–6 heptad repeats, and each residue in the heptad is labeled *a‐g*. Residue *d* is normally a leucine, and residue *a* is normally aliphatic or non‐polar; together they form the hydrophobic core of the coiled coil (Figure 1b,c). Residues *e* and *g* are usually polar or charged residues, and they interact to stabilize or destabilize the dimer interface, either side of the hydrophobic core (Figure 1b,d). Variation within these heptad repeats dictates the ability of different monomers to dimerize (Vinson et al., 2002, 2006). Fos and Jun are a canonical example of this.

The Fos and Jun families include FosB, c‐Fos, JunB, c‐Jun, and JunD. These proteins have been used as textbook examples of coiled‐coil structure motifs for decades (Branden & Tooze, 1991). It is well documented that Fos and Jun form stable heterodimers, and Jun can homodimerize, but Fos cannot form stable homodimers due to repelling charges within the L‐zip dimer interface (Branden & Tooze, 1991; Turner & Tjian, 1989).

As shown in Figure 1d,e, the *g* position residues of FosB are mostly glutamates, which interact with the positively charged *e* position residues of JunD. The favorable electrostatic interaction allows a stable dimer to form. Conversely, electrostatic repulsion between these same *g* positioned glutamates and those in position *e*, prevents FosB from forming stable homodimers (Figure 1f) (Chen et al., 2012; Vinson et al., 2006). Crucially, some dimer pairs are possible, and others are forbidden, dictated by attractive or repulsive electrostatic interactions. Being able to accurately discriminate functionally relevant L‐zip interactions from those that are forbidden has the potential to inform our understanding of the transcriptional reprogramming that occurs in cancers and in response to certain intracellular pathogens (Eferl & Wagner, 2003; Gazon et al., 2012; Krämer et al., 2015; Kuhlmann et al., 2007). The ability to predict L‐zip dimer structures accurately using AI would give us a new approach to analyze numerous different theoretically possible dimer interfaces.

In 2020, the transformer‐architecture based software AlphaFold2 (Jumper et al., 2021) was shown to predict highly accurate monomeric protein structures in the 14th Community Wide Critical Assessment of Techniques for protein Structure Prediction (CASP14). AlphaFold2 was released publicly in July 2021, and in March 2022, additional training on oligomeric protein structures was used to predict the structure of multimeric proteins with known stoichiometry (sometimes called AlphaFold Multimer). (Evans et al., 2022) Subsequent improvements were made by AlphaFold3 in May 2024, which allows for the inclusion of nucleic acids, post‐translational modifications and small ligands. (Abramson et al., 2024).

The AlphaFold2 architecture is comprised of two major modules. The Evoformer module processes input data which is used by the structure module to produce an output 3D structure. The overall model uses two sources of data to make protein structure predictions: co‐evolutionary information derived from multiple sequence alignments (MSA) and structural templates derived from the PDB. In AlphaFold2 and AlphaFold3, the proximity of protein residues is implicitly represented by a 2D residue proximity graph. This graph does not explicitly account for other effects, for example electrostatics, that can modify residue proximity (Abramson et al., 2024; Evans et al., 2022; Jumper et al., 2021). We used FosB and JunD as a test case to investigate the capabilities and limitations of AlphaFold2 when positional information for residues is only one factor to take into account. In addition, we used experimental data from a protein dimerization array performed by Newman and Keating (2003), that included 49 human L‐zip proteins, and compared the experimental results to predictions using Boltz‐1 (an unrestricted AlphaFold3 reimplementation) to test the current state‐of‐the‐art deep learning methods' ability to accurately predict L‐zip dimers at scale.

## 2 RESULTS

### 2.1 AlphaFold2 predicts FosB leucine zipper homo‐ and heterodimer structures with indistinguishable confidence

AlphaFold2 (Jumper et al., 2021) predicted the FosB monomer had an *α*‐helix covering residues P152‐G221. The rest of the protein was unstructured. For the JunD monomer, AlphaFold2 predicted two *α*‐helices: V128‐A165 and M263‐S334. (Supplementary Figure 1) It is notable that AlphaFold2 predicts these structured helices in FosB and JunD in the absence of their binding partners. Previously, it was thought that L‐zip dimer pairs formed their secondary structure only in the presence of DNA (Patel et al., 1990; Weiss et al., 1990). More recently, evidence has been found that FosB and JunD can retain their secondary and quaternary structures in the absence of DNA (Yin et al., 2017). To date, it remains unclear if all AP‐1 transcription factors maintain their secondary structure in the absence of DNA in vivo.

**FIGURE 2:**
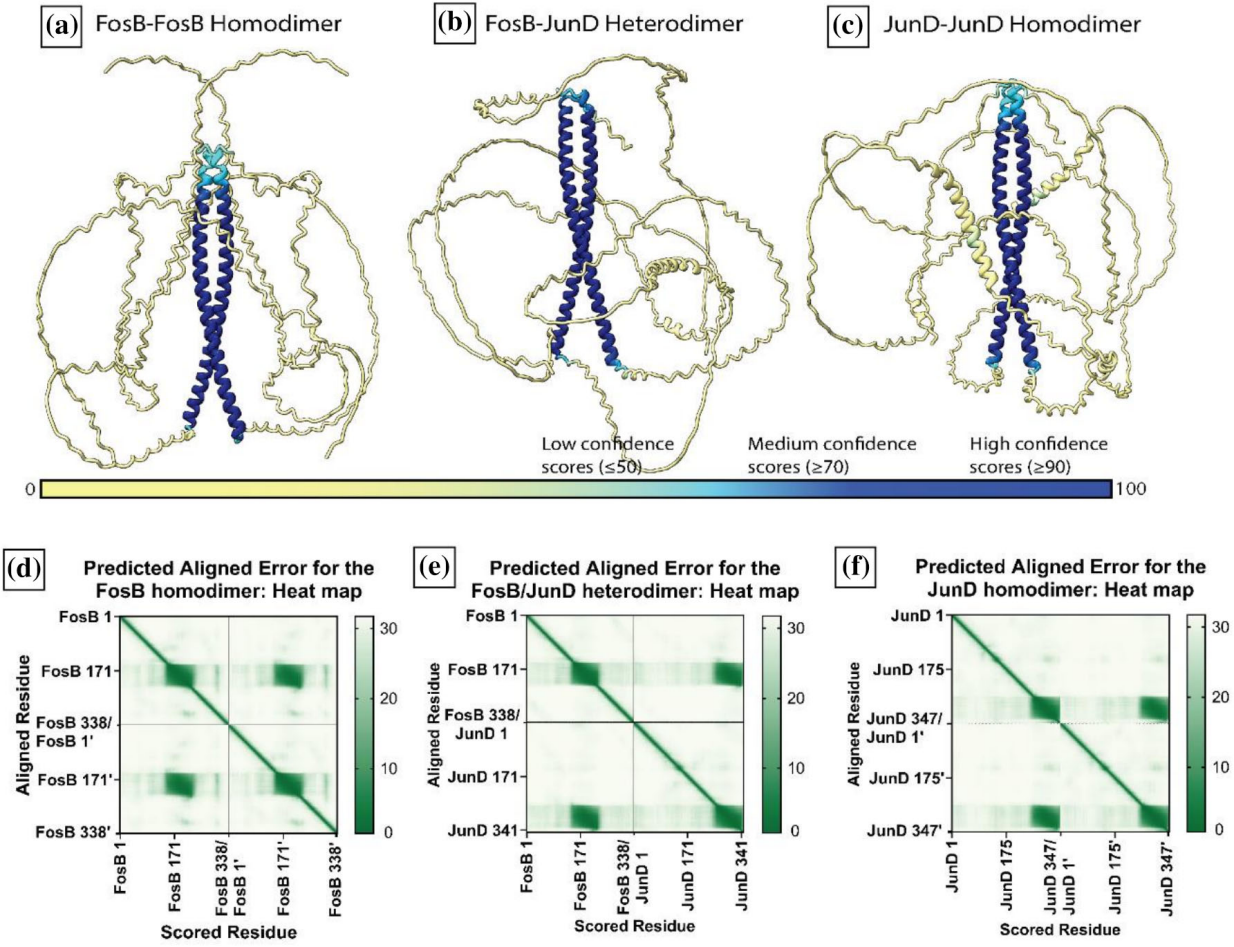
AlphaFold‐Multimer predicted structures colored by confidence score and the corresponding predicted alignment error (PAE) plots. (a) The predicted structure of the FosB homodimer colored by confidence score; (b) The predicted structure of the FosB‐JunD heterodimer colored by confidence score; (c) The predicted structure of the JunD homodimer colored by confidence score; (d) The PAE scores for the FosB homodimer shown in a heatmap; (e) The PAE scores for the FosB‐JunD heterodimer shown in a heatmap; (f) The PAE scores for the JunD homodimer shown in a heatmap.

AlphaFold2‐Multimer (Evans et al., 2022) predicted the FosB/JunD heterodimer (Figure 2b) contained a high confidence coiled‐coil dimer interface. The predicted structures of the FosB (Figure 2a) and JunD (Figure 2c) homodimers had very similar predicted structures. The coiled coil structured regions covered residues 152 ± 1–219 ± 1 of FosB and 263 ± 1–334 ± 1 of JunD. These results are consistent with the crystal structure of the FosB/JunD heterodimer (PDB code: 5VPF), which contains only residues 153–217 and 267–330, respectively. Upon first inspection, the AlphaFold2‐Multimer predicted structures of the FosB and JunD homodimers showed remarkably little difference to the FosB‐JunD heterodimer.

Alignments between the crystal structure of the FosB‐JunD heterodimer (PDB code: 5VPF) or the Jun‐Jun homodimer (PDB: 2H7H), and the three different AlphaFold2‐Multimer constructs confirmed that the coiled‐coil regions are accurate representations of an L‐zip. Within all three dimers, the L‐zip Leu (*d*) residues and the *a* residues are positioned correctly to form a hydrophobic core between the coiled‐coils. (Supplementary Figure 2) The RMSD for these alignments are all <1 Ångströms (Å) (see Table 1).

**TABLE 1:** RMSD of structural alignments for the L‐zip domain.

Dimer alignments	RMSD
Crystal_FosB‐JunD:AlphaFold_FosB‐JunD	0.679 Å
Crystal_FosB‐JunD:AlphaFold_JunD‐JunD	0.864 Å
Crystal_FosB‐JunD:AlphaFold_FosB‐FosB	0.935 Å
Crystal_FosB‐JunD:AlphaFold_Synthetic Peptide	0.984 Å
Crystal_2H7H_Jun‐Jun:AlphaFold_JunD‐JunD	0.334 Å

AlphaFold‐style methods calculate a predicted Local Distance Difference Test (pLDDT) score for each residue in the predicted structure. These pLDDT scores range from 0 to 100. A high pLDDT score is indicative of accurate local geometry within a protein chain (Jumper et al., 2021; Mariani et al., 2013). pLDDT scores of >90 are considered to be high confidence, and any score under 70 is considered to be low to very low confidence (Jumper et al., 2021; Mariani et al., 2013). Low pLDDT scores are often an indicator of intrinsically disordered domains (Jumper et al., 2021; Mariani et al., 2013).

The pLDDT scores for the structured L‐zip regions for the predicted dimer structures were all very high: ≥90. The mean pLDDT score for the FosB(T180‐V214)‐JunD(I293‐K327) heterodimer L‐zip region was 97.7, for the JunD(I293‐K327) homodimer it was 94.9, and for FosB(T180‐V214) homodimer it was 94.6 (see Supplementary Table 1 for pLDDT scores by residue). These high pLDDT scores indicate that AlphaFold2‐Multimer is highly confident in the predicted structure of all three of these coiled‐coil L‐zip regions. Large regions of the predicted FosB and JunD dimer structures had no recognizable secondary structure and very low pLDDT scores of 20–40; it is reasonable to infer that these regions are intrinsically disordered based on experimental evidence (Kumar et al., 2022; Yin et al., 2019).

**FIGURE 3:**
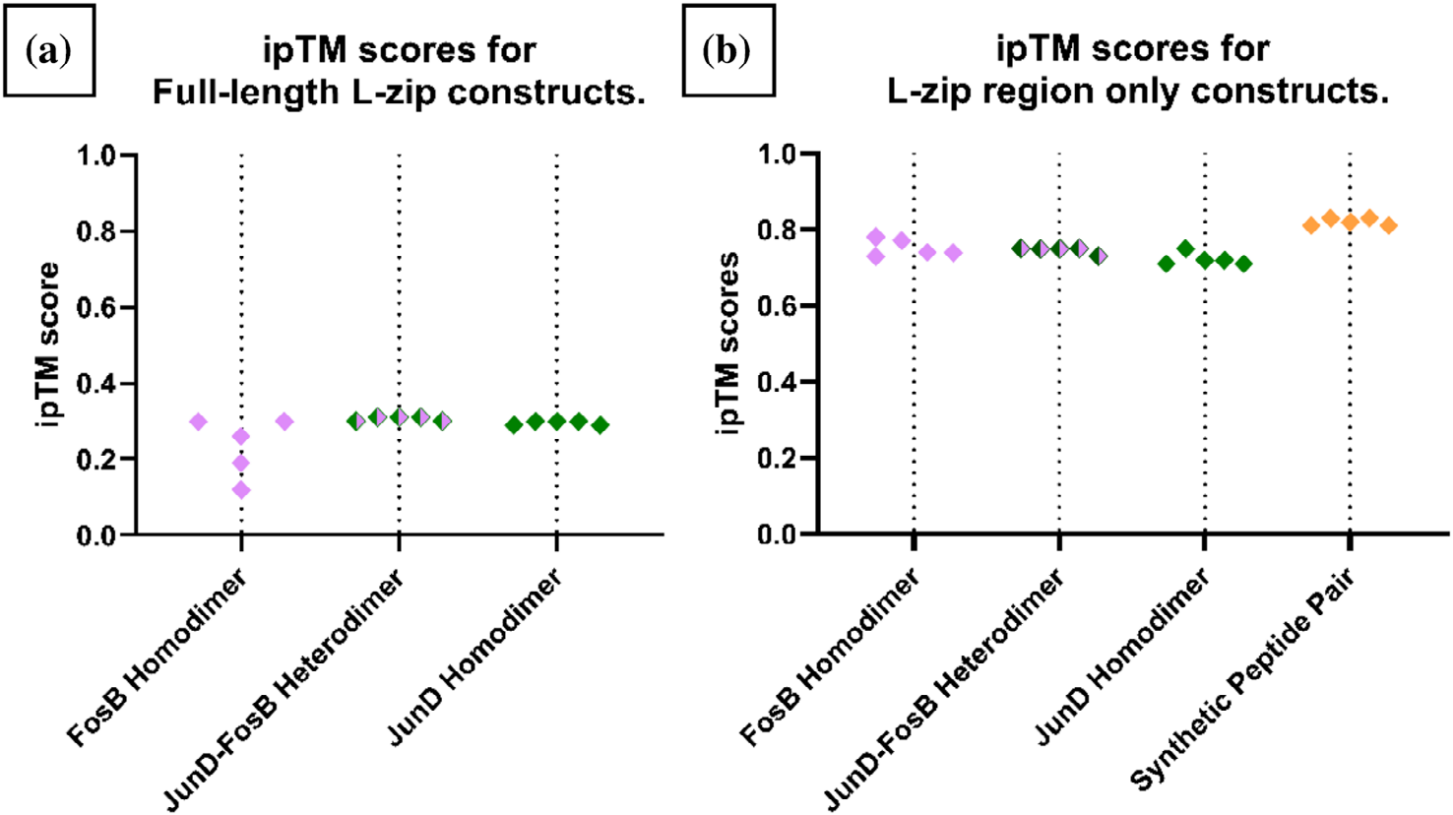
ipTM scores for the top five ranked predicted structures for each dimer construct. (a) ipTM scores for the full‐length FosB/JunD dimer pairs. (b) ipTM scores for the L‐zip region only of FosB(T180‐V214) and JunD(I293‐K327), and the synthetic peptides pair.

AlphaFold2‐Multimer can produce an “interface predicted Template Modelling” (ipTM) score, ranging from 0 to 1, for each ranked predicted complex. ipTM scores only consider residues that are not within the same chain and are used to assess the likelihood of a genuine protein–protein interaction. In general, ipTM scores ≥0.8 indicate a high confidence predicted complex, and ipTM scores of between 0.6 and 0.8 indicate medium confidence (Basu & Wallner, 2016; Evans et al., 2022). As can be seen in Figure 3a, the ipTM scores for the FosB and JunD homo/heterodimers were all low, at approximately 0.3. This was true for the dimers that are known to form in vivo (FosB/JunD and JunD/JunD), as well as the dimer unlikely to form in vivo (FosB/FosB).

We hypothesized that the low ipTM scores calculated for FosB and JunD homo/heterodimers were due to the majority of these protein complexes being intrinsically unstructured. We repeated the experiment using truncations of the proteins including just the predicted L‐zip structured regions (residues T180‐V214 of FosB and residues I293‐K327 of JunD). The ipTM scores for these L‐zip only regions were strikingly higher than the full‐length constructs, ranging from 0.7 to 0.8 for all three dimers, including the FosB homodimer (Figure 3b) Thus far, AlphaFold2‐Multimer appears to be unable to distinguish between L‐zip constructs that are likely or unlikely to form in vivo.

Predicted alignment error (PAE) scores are a measure of confidence in the relative position of pairs of residues within the predicted structure. Therefore, PAE scores can be used to assess the quality of the positioning of different domains within the predicted structure, or between monomers in multimeric predicted structures (Elfmann et al., 2023). PAE scores are measured in Å and are defined as the expected position error at residue *x* if the actual and predicted structures are aligned at residue *y*. A low PAE indicates high confidence in the relative positions of a pair of residues, and a high PAE indicates low confidence (Elfmann et al., 2023; Evans et al., 2022; Varadi et al., 2021).

The most common way to view and interpret PAE scores is through heatmaps like those in Figure *2*d–f. Each residue in the full multimer predicted structure is numbered down the *x* and *y*‐axis, creating a matrix of every possible pair of residues within the full predicted structure. The diagonal line seen down the center of each heatmap shows where every residue is aligned against itself, and the PAE score is always low, by definition (Elfmann et al., 2023; Evans et al., 2022; Varadi et al., 2021).

As can be seen in Figure 2e, the PAE score heatmap for the FosB‐JunD dimer contained two areas of low PAE scores for intra‐chain residue pairs (these can be seen along the diagonal), and two areas of low PAE scores for inter‐chain residue pairs (seen off the diagonal). The inter‐chain residue pairs exclusively fell within the L‐zip region of the FosB‐JunD dimer. This was to be expected for a well‐characterized dimer like Fos and Jun. Similar areas of low PAE scores for inter‐chain residue pairs could be seen for the JunD homodimer and the FosB homodimer (Figure 2d,f). Ordinarily, areas of low PAE scores like this would indicate an accurately predicted structure (Elfmann et al., 2023). It was remarkable that AlphaFold‐Multimer still predicted low PAE scores for the L‐zip region of the FosB homodimer when it has been shown that FosB cannot stably homodimerize in vivo (Newman & Keating, 2003; Vinson et al., 2006).

### 2.2 Electrostatic surface potentials indicate a highly unlikely dimer interaction in the FosB homodimer

Lastly, we wanted to visualize the interaction between the charged sidechains in our different dimer constructs. We did this by calculating the electrostatic surface potential of each individual α‐helix in the L‐zip constructs and examined how each charged surface interacted with its respective partner.

**FIGURE 4:**
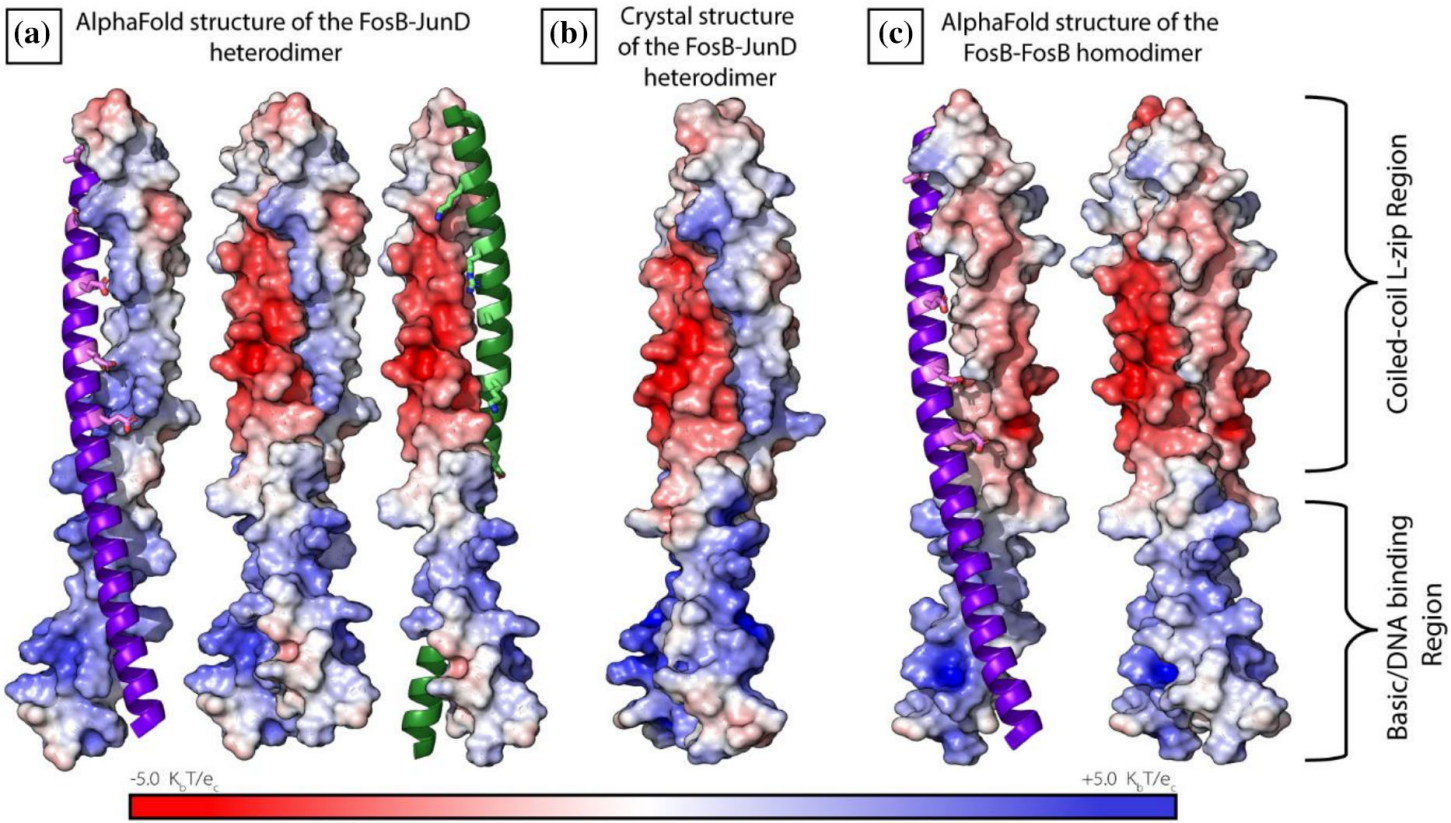
Electrostatic surface potential of the structured coiled‐coil region of: (a) the AlphaFold‐Multimer FosB‐JunD heterodimer; (b) the crystal structure FosB‐JunD heterodimer, and (c) the AlphaFold‐Multimer FosB homodimer. Throughout: FosB shown in purple, and the g positioned residues are in violet and displayed as sticks. JunD is shown in green, and the e positioned residues are in lime green and shown as sticks.

The electrostatic surface potential of the AlphaFold2‐Multimer FosB/JunD heterodimer showed that the JunD L‐zip *e* position residues formed a ridge of net‐positive charge along the *α*‐helix. The negatively charged *g* residues on FosB interact with this positively charged surface favorably (Figure 4a). These opposingly charged surface potentials recapitulated what was seen in the crystal structure of the FosB‐JunD heterodimer (Figure 4b): the two helices forming opposingly charged ridges that favorably interact.

The predicted FosB homodimer surface electrostatics told a different story. As can be seen in Figure 4c, the *e* residues of FosB created a less favorable surface potential for binding with the negatively charged FosB *g* residues. Consequently, the predicted structure of the FosB L‐zip homodimer contained two negatively charged surfaces in close proximity. There was no indication in the pLDDT scores, the PAE scores or the ipTM scores that the FosB homodimer would contain such an implausible dimer interface. When in fact, these clashing negative surface potentials are strong indicators that this dimer construct should not be able to form in vivo.

The electrostatic surface potential of the JunD homodimer was less clear. JunD had a neutral to basic overall surface potential for its coiled‐coil region (Supplementary Figure 3), without a clear pattern of opposing or repelling charges. It has been shown experimentally that JunD can homodimerize in vitro (Newman & Keating, 2003), and these results are consistent with that.

Thus far, we have shown that AlphaFold2‐Multimer can recognize and accurately predict the structures of L‐zip dimers. We have also shown that AlphaFold2‐Multimer does not clearly differentiate between probable and improbable L‐zip dimer interfaces and therefore produced high‐confidence predicted structures of the FosB homodimer, despite previous studies showing that FosB was unlikely to homodimerize (Newman & Keating, 2003; Vinson et al., 2006).

### 2.3 An energetically unfavorable synthetic peptide pair still forms a L‐zip dimer

**FIGURE 5:**
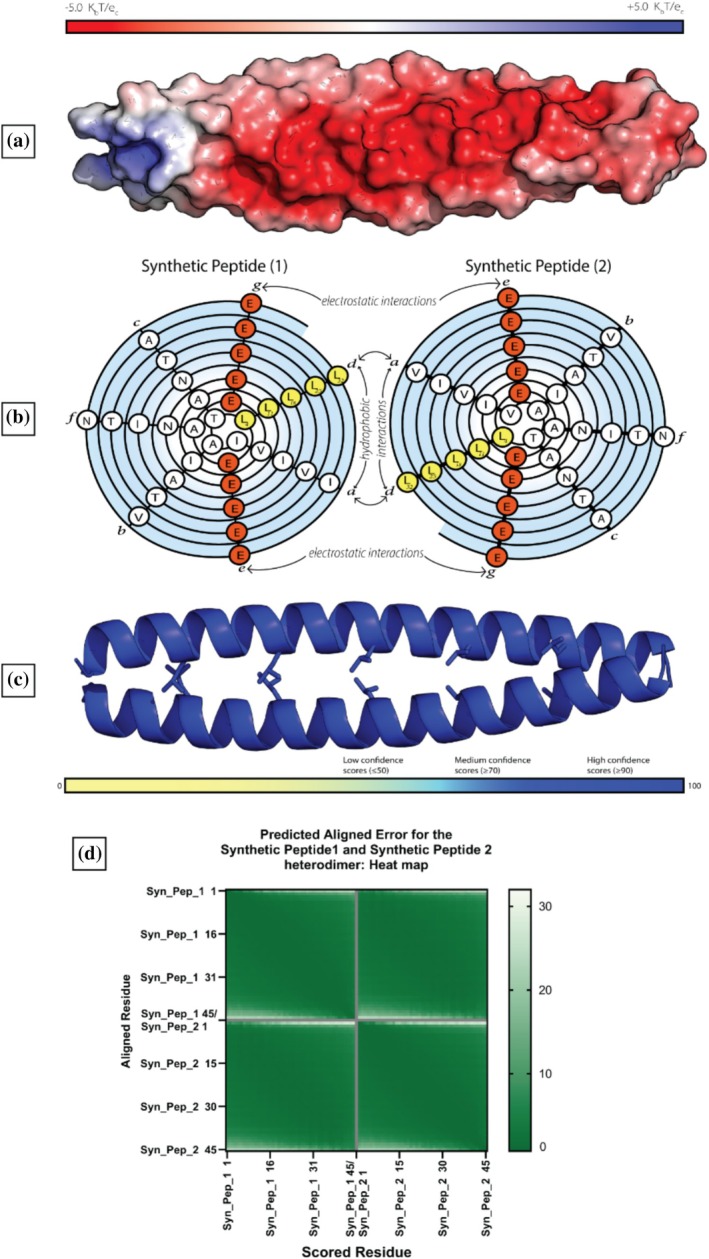
Surface potential, spiral diagram of the sequence, and predicted structure of the synthetic L‐zip peptide pair. (a) Surface of the predicted synthetic peptide colored by surface electrostatic potential. (b) Primary sequence in a spiral diagram, negatively charged residues are shown in red and Leu residues are shown in yellow. (c) Predicted structure with Leu residues shown as sticks, colored by confidence scores. (d) The Predicted Alignment Error plot.

Next, we tested if AlphaFold2‐Multimer would form a L‐Zip from a synthetic peptide, specifically engineered to be as energetically unfavorable as possible. Vinson et al. (2002, 2006) calculated the changes in coupling energy (ΔΔΔ*G*_int_) of common *g–e* pairs, relative to an Ala–Ala pair, and their data were used as a guide to design, in silico, two peptides with the highest possible coupling energy. Each peptide contained five heptad repeats, and the *e* and *g* positions were filled with repelling negatively charged residues. The first iteration of this peptide contained all Glu residues. (Figure 5b) (see Supplementary Figure 1 for the predicted monomer structures).

It should be noted that de novo protein design using AlphaFold can be difficult and is an ongoing area of research (Goverde et al., 2023). The results from the following experiments show some of the problems associated with using AlphaFold2‐Multimer to predict synthetic protein structures.

Despite the egregious clashing charges, AlphaFold2‐Multimer formed a L‐Zip dimer from these synthetic peptides. An alignment between the synthetic peptide and the FosB‐JunD crystal structure, showed that the predicted dimer structure had been formed with coiled‐coil L‐zip geometry: RMSD: 0.984 Å (Table 1 and Supplementary Figure 2). This predicted dimer structure has very high pLDDT scores (mean: 96.6); very low PAE scores (Figure 5a,c,d); and the highest ipTM score is 0.83. In fact, the ipTM scores for our synthetic peptide pair were higher than those for the FosB/JunD heterodimer (L‐zip region only) (Figure 3b). Looking at the AlphaFold confidence metrics, it appeared that there was no way to distinguish the dimerization propensity of our synthetic peptide pair from that of the FosB‐JunD dimer; both predicted structures had excellent confidence scores, but drastically different chances of forming in vivo.

### 2.4 Re‐running AlphaFold Multimer without MSA and without structural templates does not help differentiate between probable and improbable L‐zip dimers

AlphaFold2‐Multimer uses two input data sources to perform protein structure predictions: MSAs to find evolutionarily related proteins using primary structures and homologous “template” protein structures from the PDB database (Evans et al., 2022; Jumper et al., 2021). To try and ascertain what in AlphaFold Multimer's workflow caused the implausible FosB dimer to have such high confidence metrics, we re‐ran the three possible FosB and JunD dimer pairs, as well as our synthetic peptide pair, with the MSA component turned off; with the template component turned off, or with both components turned off.

When AlphaFold‐2 Multimer is run without MSA, all three possible predicted FosB/JunD dimers produced strikingly similar results (Supplementary Figure 4A). The expected structured regions are parallel coiled‐coils with high pLDDT scores of ≥90. The same regions have correspondingly low PAE scores. This is true for both the full‐length predicted structures and the L‐zip only predicted structures for all three dimers. Notably, the ipTM scores for the L‐zip only predicted structures are all high, ranging from 0.72 to 0.81. This pattern of high pLDDT scores, low PAE scores and a high ipTM score is repeated with our energetically unfavorable synthetic peptide pair (Vinson et al., 2002, 2006).

When we repeated this experiment with the MSAs turned on, but without structural templates, the results are the same. The same residues are predicted to form a parallel coiled‐coil, the pLDDT scores remain high for all four dimers; the PAE scores remain low for the appropriate structured regions and the ipTM scores are low for the full‐length predicted structures and high for the L‐zip only and synthetic predicted structures (Supplementary Figure 4A).

These results show that removing the MSAs or the templates from the calculation made no meaningful difference to the output predicted L‐zip structures, and AlphaFold was still unable to discriminate between favorable and unfavorable dimer pairs.

**FIGURE 6:**
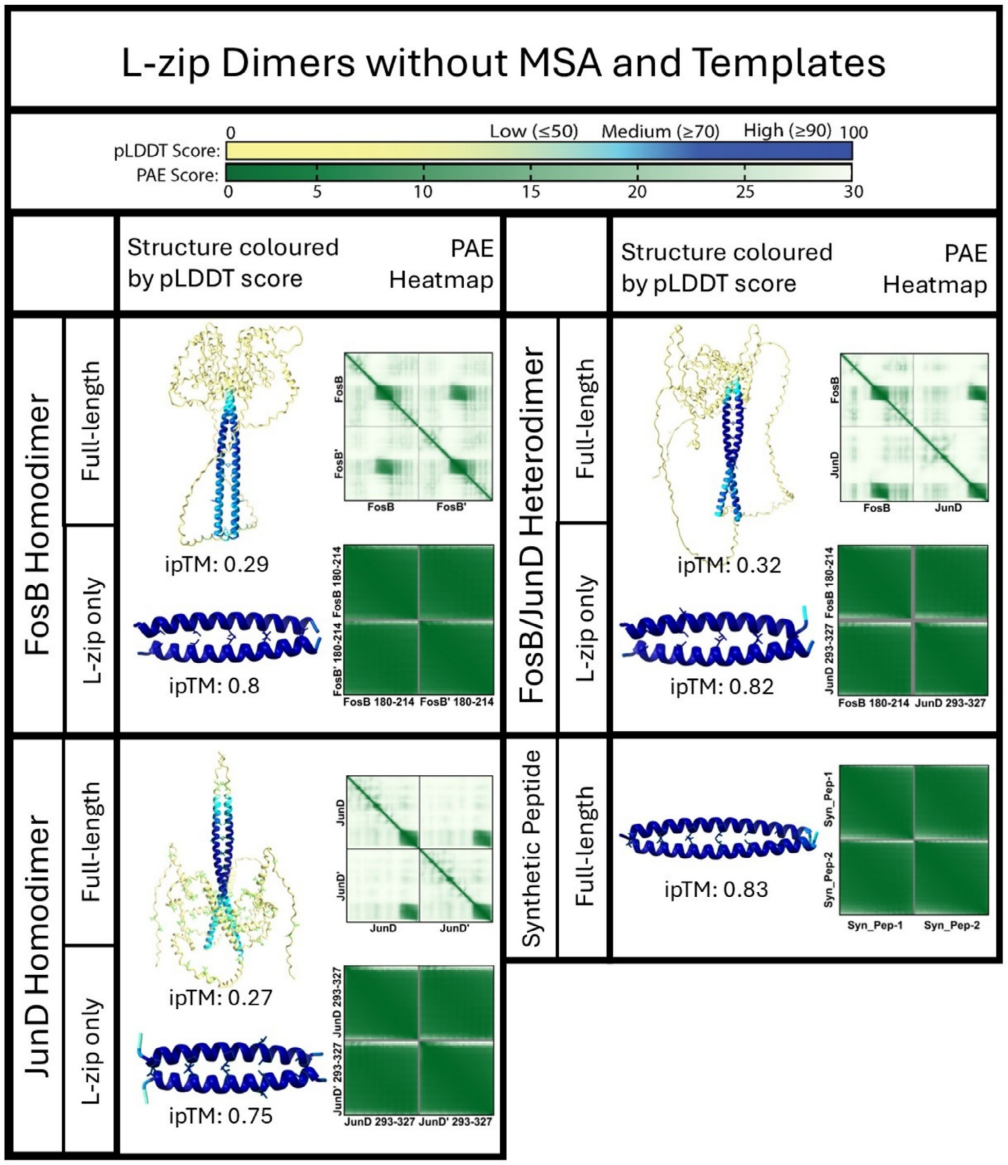
An overview of the results of modeling the three possible FosB/JunD dimers in AlphaFold2 both without MSA and without structural templates (Colabfold v1.5.5). The predicted structure for the full‐length proteins and an L‐zip region only (FosB: T180‐V214 and JunD: I293‐K327) is shown colored by pLDDT score. The corresponding PAE scores are shown as a heatmap next to each predicted structure. An ipTM score is shown for each predicted structure.

We repeated this experiment a third time without templates and the MSA turned off. We found that disabling both these elements at the same time produced nearly identical results to those previously described (see Figure 6). Conspicuously, the synthetic peptide pair, specifically designed with egregious clashing charges, had the highest ipTM score of all four dimers, at 0.83.

In our experiments, running AlphaFold‐2 Multimer without MSA, without structural templates, or without both still produced highly confident results for the FosB homodimer and our synthetic peptide pair, indicating that none of these techniques can be definitively used to differentiate functionally relevant charge interactions from prohibited interactions (see supplementary Figure 5 for AlphaFold3 predictions).

### 2.5 Analysis of an array of validated human L‐zip proteins shows a high false positive rate of dimer formation

To explore whether the observations from the JunD:FosB system were generalizable to other L‐zip proteins, we decided to investigate if AlphaFold was able to distinguish between stable and unstable L‐zip dimer pairs on a much larger collection of data. Newman and Keating (2003) performed protein dimerization arrays using the L‐zip domains from 49 human L‐zip proteins to determine which protein pairs were more or less likely to dimerize. These arrays included AP‐1 transcription factors like FosB (Supplementary Figure 6) and JunD, but also included a much larger selection of transcription factors that bind DNA via a L‐zip dimerization domain (Figure 1a.) Using the data from Newman and Keating (2003) we were able to rank 1928 possible L‐zip pairs, from 1 to 6, by their likelihood to dimerize, which we refer to as the “Newman Rank.” For the purposes of data analysis, we have considered any rank of 4 or higher as likely to dimerize (*n* = 286), and any rank of 3 or less as unlikely to dimerize (*n* = 1642). Dimer pairs that had insufficient or inconsistent data, as reported by Newman and Keating (2003), were excluded from our analysis.

Using the residue sequences reported in their Supplementary Material as input data (Newman & Keating, 2003), we used AlphaFold v2.3.2 to predict all the potential dimer pairs. Examination of the structures revealed 46 dimer pairs that did not form L‐zips of at least one heptad long. Of these 46 pairs, 43 (93.5%) scored 3 or below on the Newman Rank. To investigate the performance of modern diffusion‐based structure prediction models, we also ran every possible dimer combination through the Boltz‐1 software package, an academic open‐source re‐implementation of AlphaFold3 (Abramson et al., 2024; Wohlwend et al., 2024). Using Boltz‐1, 126 pairs failed to form a L‐zip structure, of which 123 (97.6%) had a Newman Rank less than 3. These results demonstrate that failure to predict an L‐zip dimer using AlphaFold is a strong indicator of an inability to dimerize in vitro.

**FIGURE 7:**
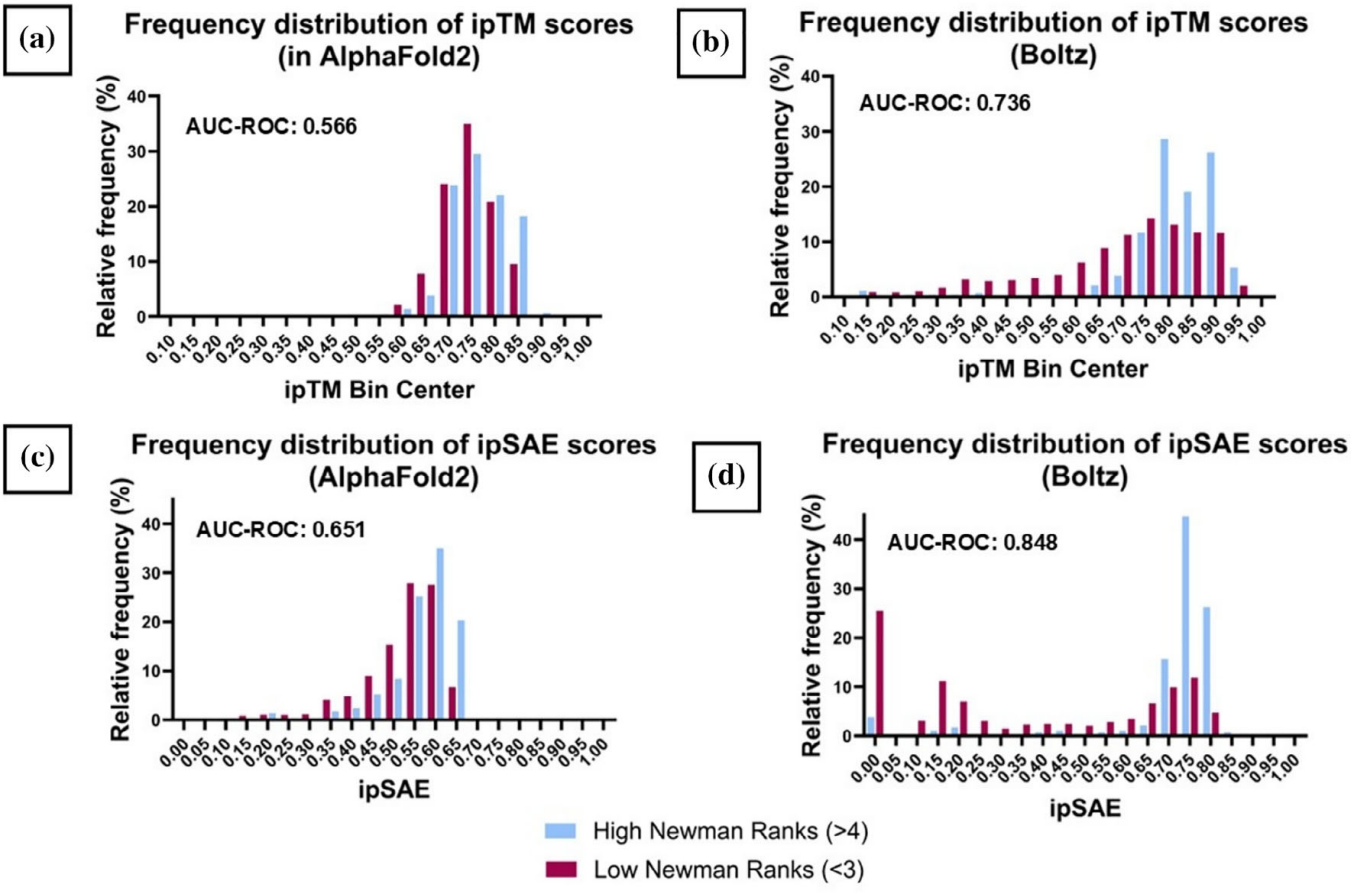
Histograms of the relative frequency of ipTM and ipSAE scores for L‐zip dimers predicted using AlphaFold2 or Boltz‐1. Throughout: “true pairs” with high Newman ranks (>4) in blue, and “false pairs” with low Newman ranks (<3) in dark red. An AUC–ROC statistic is quoted for each dataset.

For dimer pairs where L‐zip dimer structures were predicted, we assessed the ipTM score for its ability to discriminate between experimentally validated true and false dimers. By plotting the frequency distribution of the ipTM scores for our AlphaFold2 and our Boltz datasets, we can compare the distribution of predicted dimers with high Newman ranks and low Newman ranks (Figure 7a,b). For this purpose, we have split the data set into “true dimers” with a Newman rank of >4 and “false dimers” with Newman ranks of <3. Dimers predicted using AlphaFold2 with high ipTM scores have very little enrichment for true binders (Figure 7a). An AUC‐ROC analysis shows ipTM scores have almost no discrimination power (AUC‐ROC = 0.566) between true and false binders. Boltz‐1 shows some improvement on this (Figure 7b) with some difference between the distribution of true and false binders and an AUC–ROC of 0.736. While an improvement, both approaches have a high false‐positive rate (83.5% for AlphaFold2 and 63.1% for Boltz‐1) when using ipTM.

We next examined whether the mean pLDDT or mean PAE scores (produced by Boltz‐1) could be used to differentiate between true or false binders, and found that both confidence metrics had similarly high false positive rates to ipTM: 71.1% for mean pLDDT and 55.6% for mean PAE. Conversely, the false negative rate is very low for all three quality statistic: ipTM 0.6%, mean pLDDT 0.7% and mean PAE 2.0% (Supplementary Figure 7). These results indicate that, in the context of L‐zip dimers, poor confidence metrics and failed dimer predictions are highly likely to represent true non‐binders.

The phenomenon of ipTM scores being affected by unstructured regions of a predicted protein has been studied by Dunbrack (2025), who proposes the use of a new quality metric: interaction prediction Score from Aligned Errors (ipSAE). ipSAE scores effectively only include residue pairs with low PAE scores. We calculated ipSAE scores for each dimer pair in both the AlphaFold2 and Boltz‐1 datasets and plotted frequency distribution graphs for true and false L‐zip dimers (Figure 7c,d). The AUC–ROC value of 0.651 calculated for the AlphaFold2 dataset (Figure 7c) showed that this approach is of poor discriminatory potential, with the distribution of scores very similar between high and low Newman Rank dimers. However, the Boltz‐1 approach yielded an AUC‐ROC value of 0.848 (Figure 7d). In this dataset, the high Newman rank dimer pairs skewed to high ipSAE scores, while low Newman rank pairs had a bimodal ipSAE score distribution, improving the probability of identifying true negatives. Nevertheless, false positives remained approximately 1 in 3.

### 2.6 L‐zip dimer prediction is dominated by leucine patterning and the commonness of *e* and *g* position residues, not electrostatics

As a final experiment, we wanted to test what would “break” the L‐Zip dimer by using the synthetic peptide pair as a backbone and changing either the *e/g* residues, or the Leu (*d*) residues. These protein structure predictions were done using Galaxy Australia's AlphaFold Service (Galaxy Community, 2024). Either all the *e/g* residues were changed to the same charged residue, or one, two or three Leu residues were changed to Ile residues. A predicted L‐zip construct was recorded as “broken” when it included anti‐parallel helices, parallel helices with incorrectly oriented Leu residues, or helices with very minimal interchain interaction (see Table 2). It should be noted that the different permutations of our synthetic peptide pair listed in this experiment are based on general trends in L‐zips as described by Vinson et al. (2002) and (2006) rather than experimental data.

**TABLE 2:** Results of synthetic L‐zips in AlphaFold2‐Multimer (using Galaxy Australia's AlphaFold Service (Galaxy Community, 2024)).

Charged *e/g* residues						1× Leu to Ile						2× Leu to Ile						3× Leu to Ile					
Neg *e/g* E	Y	Y	Y	Y	Y	ILLLL	N	N	Y	Y	Y	IILLL	N	N	N	N	Y	ILLLL_LIILL	N	N	N	N	N
Neg *e/g* D	N	N	N	N	N	LILLL	N	N	Y	Y	N	ILLLL_LILLL	N	N	N	N	N	ILLLL_LLLII	N	N	N	Y	Y
						LLILL	Y	Y	Y	Y	Y	ILILL	N	N	N	N	N	ILLLL_IILLL	N	N	N	N	N
Pos *e/g K*	Y	Y	Y	Y	Y	LLLIL	Y	Y	N	Y	N	LILLL_LLLIL	N	N	N	Y	Y	LILLL_LIILL	N	N	N	N	N
Pos *e/g* R	N	N	N	Y	N	LLLLI	Y	Y	Y	Y	Y	LLILL_LLILL	N	Y	N	Y	N	LILIL_LLILL	N	Y	N	Y	Y
												ILLLL_LLLLI	N	N	N	N	Y						
												ILLLI	N	Y	N	Y	Y						

*Note*: 1×, 2×, and 3× refer to the number of Leu to Ile mutations incorporated into the synthetic peptide pair. Any synthetic peptide pair that produced a dimeric coil–coil L‐zip predicted structure in AlphaFold2‐Multimer was marked as Y for Yes. Any peptide pair that produced any other predicted structure in AlphaFold‐Multimer was marked N for No. AlphaFold‐Multimer was set to produce five ranked predicted structures, each predicted structure was assessed separately, producing five Y or N scores for each Synthetic peptide pair (see Supplementary material for PDB files).

Placing Glu or Lys residues in all the *e/g* positions still produced L‐zips in all five AlphaFold2‐Multimer predictions. However, Asp and Arg *e/g* residues “broke” the L‐zip and caused AlphaFold2‐Multimer to produce alternative dimer interfaces. Sequence alignments of human L‐zip proteins show that Arg and Asp are less commonly found in the *e/g* position than Lys or Glu (Vinson et al., 2002), and this could explain why AlphaFold2‐Multimer stopped recognizing the L‐zip heptad repeats when so many uncommon residues were included in the primary structure. Changing one Leu to an Ile mostly maintained the L‐zip construct, whereas progressively mutating the Leu residues to Ile increased the number of “broken” L‐zip constructs (see Table 2). From these data there is no obvious single Leu position whose mutation consistently results in a “broken” L‐zip dimer, as predicted by AlphaFold2‐Multimer. There is, however, a trend that increasing the number of Leu to Ile mutations at the *d* position results in increasingly fewer predicted L‐zip dimers. These findings indicate that the principal factor driving L‐zip dimer formation in AlphaFold is the heptad repeating nature of Leu residues at position *d* while being relatively agnostic to the influence of functionally critical electrostatics.

## 3 DISCUSSION

The neural network at the heart of the various AlphaFold iterations builds and progressively refines a representation of the proximity of protein residues derived from evolutionary constraints (Evans et al., 2022; Jumper et al., 2021). The evidence presented in this study demonstrates that AlphaFold does not fully account for other biophysical effects (e.g., electrostatics) that can modify proximity. Additionally, the relaxation algorithms utilized by AlphaFold2 and AlphaFold‐Multimer (with the Amber ff99SB force field) are strictly downhill, meaning they cannot counteract the large scale destabilizing electrostatic interactions found in our pre‐constructed AlphaFold L‐zip dimers (Bouatta et al., 2021; Hornak et al., 2006; Jumper et al., 2021). L‐zips are an excellent demonstration of these limitations: AlphaFold can accurately identify the distinctive L‐zip regions, without recognizing when clashing charges make the predicted dimer structure improbable.

It should be noted that deep learning/artificial intelligence based protein structure modeling is a rapidly evolving field. At the time of writing, AlphaFold3 is available for the general public to use and the advances in model architecture have been utilized by the open‐source software Boltz‐1 (Abramson et al., 2024; Wohlwend et al., 2024). Of the various iterations of AlphaFold that we used in this study, which included removing the structural templates or the MSA component, none showed a clear ability to reliably differentiate different classes of L‐zip dimers (Supplementary Figures 4 and 5). The combination of Boltz‐1 and ipSAE scores did show a marked improvement on the other approaches, with strong negative prediction powers (Figure 7d). However, the high false‐positive rate in all approaches, including the Boltz‐1/ipSAE combination, demonstrates that further screening techniques are needed to accurately discriminate between true and false L‐zip dimer predictions.

The points raised in this study are important to consider when using AlphaFold to study any protein that forms homodimers or heterodimers as part of a combinatorial control network, or any proteins that form coiled‐coil dimers. As a specific example, disruptions to the network of AP‐1 transcription factors have been implicated in the development of bone, skin, liver, and lung cancer, to name but a few (Eferl & Wagner, 2003). Assessing the different possible interactions within this nuanced network of transcription factors would be challenging using even the most up‐to‐date AlphaFold iterations. Similarly, to use AlphaFold to design artificial dimers would require the use of additional data or software to evaluate binding partner specificity and affinity.

In the case of L‐zips, previous experimental data can be used to evaluate AlphaFold structures. The coupling energies experimentally calculated by Vinson et al. (2002, 2006) can be used to estimate if a L‐zip dimer will be stable. Unfortunately, Vinson et al. (2002, 2006) only tested the most commonly found residues in these positions; therefore, this approach could not be used to accurately calculate the coupling energy of the FosB‐JunD dimer because JunD contains a relatively rare Thr as one of its *g* residues. However, previous experimentation like this can, and should, be used to assess the quality of AlphaFold structures.

It should be emphasized that AlphaFold does not provide a theoretical K_D_ for predicted structures, or any other indication of binding strength, which is an important consideration in systems like this that rely on relative affinities to exert combinatorial control. For example, we have assumed in this study that FosB cannot dimerize in any form. However, a truncated FosB variant (ΔFosB), lacking the C‐terminal 101 residues but still containing the L‐zip domain, accumulates in mouse brain tissue (Nestler, 2015) and may homodimerize at high concentrations to act as an aberrant AP‐1 transcription factor (Jorissen et al., 2007). While an experimental ΔFosB homodimer structure can be found in the PDB (PDB ID: 6UCM), this structure displays an antiparallel helical conformation which is inconsistent with the AlphaFold prediction which adopts a classical parallel coiled‐coil L‐zip with high confidence. To date, it remains unclear what the biologically relevant oligomeric structure of ΔFosB is, and evidence indicates that ΔFosB will only dimerize at a concentration that is higher than the normal cellular concentrations of full length FosB (Jorissen et al., 2007; Yin et al., 2020). It is theoretically possible that AlphaFold is predicting an accurate, but extremely weak binding FosB homodimer pair, with no indication that this dimer should not form under normal cellular conditions.

The evidence presented in this study suggests that current deep learning models for protein structure prediction are not well suited to distinguish between functionally relevant and irrelevant L‐zip dimer pairs.

Altogether, this study is an important example of a class of proteins for which AlphaFold predicts structures with high confidence but low accuracy. It illustrates some of AlphaFold's strengths and weaknesses, showing that AlphaFold structures must be interpreted critically and with prior knowledge of the physical principles affecting protein 3D geometry.

## 4 METHODS

Unless otherwise stated, AlphaFold2 was accessed using ColabFold v1.5.5 (Mirdita et al., 2022). The msa_mode used was “mmseqs2_uniref_env”; the model_type used was “alphafold2_multimer_v3” and the template_mode used was “pdb100.” All other settings were left as default. The top ranked model was used for all further analysis unless stated otherwise. To run AlphaFold2 without MSAs, the msa_mode was set to “single_sequence.” To run AlphaFold2 without templates, the template_mode was set to “none”. All confidence metrics, including ipTM scores, pLDDT scores, and PAE scores were extracted from the top ranked .JSON file for each predicted structure.

This study used full length sequences of FosB and JunD, sourced from UniProt (The UniProt Consortium, 2019), to ascertain if AlphaFold2 (Evans et al., 2022; Jumper et al., 2021) can recognize the L‐zip motifs within the full sequences. FosB (UniProt ID: P53539) and JunD (UniProt ID: P17535) were input into AlphaFold2 as monomers, homodimers, and a heterodimer.

The structure alignments were calculated with the Pymol Alignment plugin using the default settings (Schrodinger LLC, 2015). (Pymol Command: extra_fit *x*, *y*, method = align, cycles = 5, cutoff = 2.0, mobile_state = −1, target_state = −1.) For each structural alignment discussed, the AlphaFold predicted structure was aligned to the target crystal structure of the FosB‐JunD heterodimer (PDB ID: 5VPF) (Yin et al., 2019) or the JunD‐JunD homodimer (PDB ID: 2H7H) (Berman et al., 2000). The L‐zip domain from the crystal structures was isolated, FosB(T180‐V214) and JunD(I293‐K327), and the corresponding residues from the AlphaFold predicted structures were also isolated and aligned to the target. For the synthetic peptide alignments, residues 6–40 of synthetic peptides 1 and 2 were isolated and aligned to the L‐zip domain, FosB(T180‐V214) and JunD(I293‐K327), of the PDB ID: 5VPF crystal structure target. Isolating the L‐zip domains removed undue influence from the unstructured/highly variable regions of the FosB/JunD predicted AlphaFold structures and ensured consistency across the different structural alignments.

The surface electrostatics were calculated using the Pymol APBS electrostatics plugin using the default settings (Jurrus et al., 2018; Schrodinger LLC, 2015).

Predicted model structures colored by pLDDT score were produced using the command “color bfactor palette 100,98,60:cyan:darkblue range 50,90” in molecular graphics software UCSF ChimeraX (Meng et al., 2023).

When required, PAE scores were imported into GraphPad Prism Version 10.2.0 (GraphPad Software) and used to make heatmaps of the PAE scores and other figures.

To predict all the possible dimer pairs of the human L‐zips as named by Newman and Keating (2003) we used open‐source deep learning model Boltz‐1, which reimplements the AlphaFold3 architecture. (Wohlwend et al., 2024) Boltz‐1 was used due to the software being able to handle large datasets at speed. The input FASTA files were generated from the residue sequences listed in the Supplementary Material of Newman and Keating's (2003) paper. These residue sequences cover the basic region and the L‐zip/coiled‐coil region of each protein. Where the specific residue sequence is stated, short C‐terminal sequences that facilitated cloning were included. N and C terminal cloning vector sequences were not included. Every possible dimer combination, including homodimers, was used to predict a dimer structure. Synonymous dimer pair structures, for example, NEF2‐BACH1 and BACH1‐NEF2, were not predicted twice. For the multiple sequence alignment, homologous sequence searching using a GPU‐accelerated build of MMseqs (commit # 1668032, no gpu‐server) (Kallenborn et al., 2024) was performed in order to rapidly generate sequence alignments for input into Boltz‐1 for all possible dimer combinations (1274 in total). Boltz‐1 v0.41 was run with default settings plus the inclusion of the ‐‐write_full_pae option. Shell scripts were written to re‐format all the associated FASTA files to point to the correct .a3m multiple sequence alignment files from MMSeqs‐GPU and to manage efficient job submission and validation.

Mean pLDDT confidence scores and ipTM values were taken from the associated confidence_[protein]_model.json files by extracting on the relevant text string. Mean PAE values were taken from the associated pae_[protein]_model.npz file with a short python script that loaded the numpy arrays, processed and reported the mean and standard error. These values were copied into a spreadsheet for further analysis. See Supplementary File L‐zip dimer pairs AlphaFold confidence metrics.xlsx for the raw data. The trend line seen on Figure 7a was calculated in GraphPad Prism Version 10.2.0 using a simple linear regression.

The “Newman Ranks” were extracted from the data produced by Newman and Keating (2003). Each dimer pair was ranked according to the Z‐scores stated in Figure 2 of the 2003 paper. A Z‐score of >20 was ranked 6; >10 was ranked 5; >5 was ranked 4; >2.5 was ranked 3; >1.5 was ranked 2; >1 was ranked 1 and any unassigned data or where there was no reciprocal signal was ranked 0 and discounted from any further analysis. Attempts to recalculate the Z‐scores from the raw data were unsuccessful. Due to the nature of the protein arrays used to generate these Z‐scores, synonymous dimer pairs, for example, NEF2‐BACH1 and BACH1‐NEF2, may have slightly different Newman Ranks. Overall, the data collected by Newman and Keating (2003) showed high symmetry, reinforcing the reliability of their experimental methods. However, due to the subtle difference seen in some synonymous dimer pairs, each synonymous dimer was analyzed separately. This means each heterodimeric predicted structure was analyzed twice for each synonymous Newman Ranked dimer. See Supplementary File L‐zip dimer pairs Newman Ranks and structures.xlsx for the raw data.

Structures of the synthetic L‐zip peptide pairs listed in Table 2 were predicted using AlphaFold2. In this case, AlphaFold2 was accessed using the Galaxy Australia platform (Galaxy Community, 2024), running AlphaFold v2.3.2. All AlphaFold computations were run using the default settings, as implemented by Galaxy Australia, with Amber ff99sb force field (Hornak et al., 2006) relaxation enabled and a full database selected. Five PDB protein structure files were produced, as per the default settings, for each AlphaFold computation.

To predict the structures of all the L‐zip dimers listed in the Newman and Keating (2003) study in AlphaFold2, we used a local deployment of AlphaFold v2.3.2 using the small BFD database to ensure the highest quality prediction possible.

AUC‐ROC statistics were calculated in GraphPad Prism Version 10.2.0 (GraphPad Software), using the ROC‐curve built‐in analysis and the default settings.

ipSAE scores as described by Dunbrack (2025) were generated using a PAE cutoff of 3 and a distance cutoff of 5.

AlphaFold3 was accessed (September 2024) via the Google DeepMind AlphaFold Server. (Abramson et al., 2024) Each L‐zip monomer primary structure was entered as a separate Protein Entity without any post‐translational modifications, ions, ligands, or nucleic acids. Full length, L‐zip only structures for the three FosB and JunD dimers were predicted, as stated previously.

## AUTHOR CONTRIBUTIONS

**Isobel Mitic:** Investigation; writing – original draft; methodology; validation; visualization; writing – review and editing; formal analysis; data curation; conceptualization. **Keiran Rowell:** Writing – review and editing; methodology; validation. **Thomas Litfin:** Writing – review and editing; validation. **Katharine A. Michie:** Writing – review and editing; conceptualization; supervision; resources; validation. **David A. Jacques:** Conceptualization; funding acquisition; writing – review and editing; supervision; project administration; resources.

## Supporting information


**Supplementary Figure 1.** AlphaFold2 predicted structures of the monomers of FosB, JunD, Synthetic Peptide 1 and Synthetic Peptide 2.
**Supplementary Figure 2**: Alignments between the crystal structure of the FosB‐JunD heterodimer and different AlphaFold2 predicted structures.
**Supplementary Figure 3**: Electrostatic surface potential of the predicted structured coiled‐coil region of the AlphaFold2 JunD homodimer.
**Supplementary Figure 4**: An overview of the results of modeling the three possible FosB/JunD dimers without MSA and templates.
**Supplementary Figure 5**: An overview of the results of modeling the three possible FosB/JunD dimers in AlphaFold3.
**Supplementary Figure 6**: The minimal L‐zip only region of the FosB homodimer structure as predicted by Boltz‐1.
**Supplementary Figure 7**: Graph for L‐zip dimer predictions from Boltz‐1 showing Newman rank against ipTM, mean pLDDT and mean PAE.
**Supplementary Table 1**: The pLDDT scores for the individual residues in the L‐zip regions of the full length AlphaFold Multimer predictions for the three possible FosB and JunD dimers.
**Supplementary material:** .zip file of all the PDB files for the synthetic peptide pairs predicted structures produced in AlphaFold‐Multimer.
File name: Synthetic Leucine zipper construct PDB files.zip.
**Supplementary material:** .zip file of all the .cif files for the human L‐zip dimers as named by Newman and Keating (2003).
File name: leucine_zippers_Boltz_predictions.zip.
**Supplementary material:** Excel file containing the confidence metrics for the predicted structures of the human L‐zip dimers as named by Newman and Keating (2003).
File name: L‐zip dimer pairs AlphaFold confidence metrics.xlsx.
**Supplementary material:** Excel file containing the Newman Rank and basic structural characterization for the human L‐zip dimers as named by Newman and Keating (2003).
**File name:** L‐zip dimer pairs Newman Ranks and structures.xlsx.

## Data Availability

The data that supports the findings of this study are available in the supplementary material of this article.
