# Supplementary material for: Assessing the validity of leucine zipper constructs predicted by AlphaFold
Source: Protein Sci. 2025 Dec 23;35(1):e70438. doi: 10.1002/pro.70438 (PMC12723748; doi:10.1002/pro.70438)
Supplement: Supplementary file 1 — Supplementary Figure 1. AlphaFold2 predicted structures of the monomers of FosB, JunD, Synthetic Peptide 1 and Synthetic Peptide 2. Supplementary Figure 2: Alignments between the crystal structure of the FosB‐JunD heterodimer and different AlphaFold2 predicted structures. Supplementary Figure 3: Electrostatic surface potential of the predicted structured coiled‐coil region of the AlphaFold2 JunD homodimer. Supplementary Figure 4: An overview of the results of modeling the three possible FosB/JunD dimers without MSA and templates. Supplementary Figure 5: An overview of the results of modeling the three possible FosB/JunD dimers in AlphaFold3. Supplementary Figure 6: The minimal L‐zip only region of the FosB homodimer structure as predicted by Boltz‐1. Supplementary Figure 7: Graph for L‐zip dimer predictions from Boltz‐1 showing Newman rank against ipTM, mean pLDDT and mean PAE. Supplementary Table 1: The pLDDT scores for the individual residues in the L‐zip regions of the full length AlphaFold Multimer predictions for the three possible FosB and JunD dimers. Supplementary material: .zip file of all the PDB files for the synthetic peptide pairs predicted structures produced in AlphaFold‐Multimer. File name: Synthetic Leucine zipper construct PDB files.zip. Supplementary material: .zip file of all the .cif files for the human L‐zip dimers as named by Newman and Keating (2003). File name: leucine_zippers_Boltz_predictions.zip. Supplementary material: Excel file containing the confidence metrics for the predicted structures of the human L‐zip dimers as named by Newman and Keating (2003). File name: L‐zip dimer pairs AlphaFold confidence metrics.xlsx. Supplementary material: Excel file containing the Newman Rank and basic structural characterization for the human L‐zip dimers as named by Newman and Keating (2003). File name: L‐zip dimer pairs Newman Ranks and structures.xlsx. [file PRO-35-e70438-s001.zip › Dec_2025__Supplementary material_Assessing the Validity of FosB-JunD Leucine Zipper Constructs Produced by Alphafold.docx]

**Title: Assessing the Validity of Leucine Zipper Constructs Predicted by AlphaFold- Supplementary Figures**

*
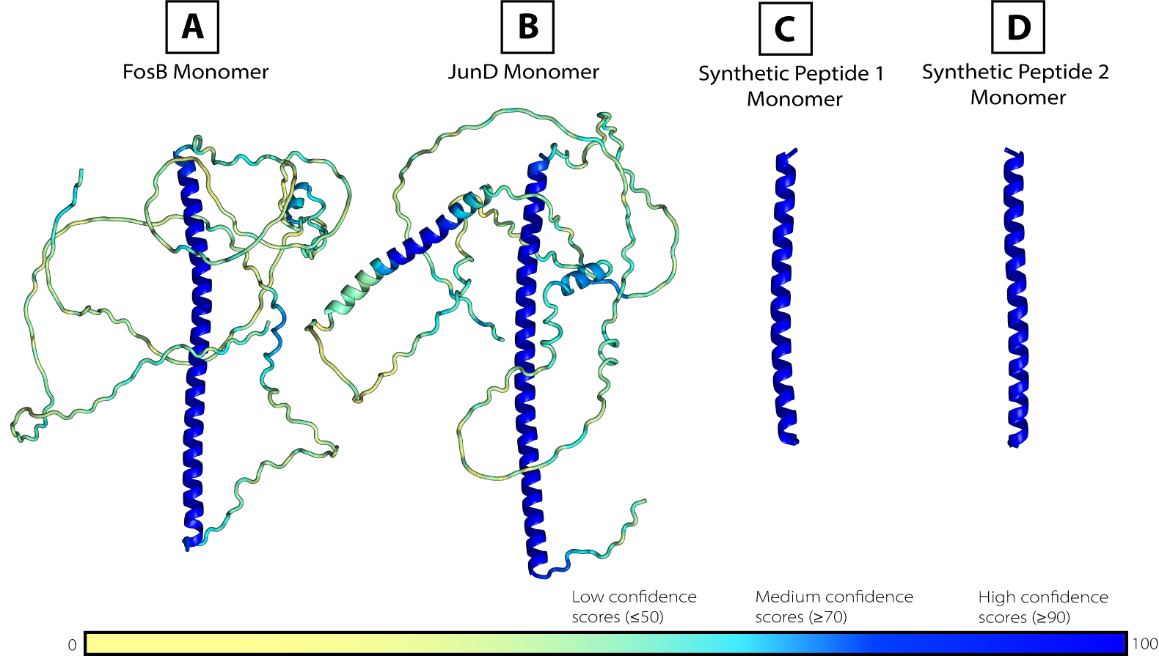
*

*Supplementary Figure 1: AlphaFold2 structures of the monomers of: A- FosB; B- JunD; C- Synthetic Peptide 1; D- Synthetic Peptide 2. All structures have been coloured by confidence score, Dark blue shows high confidence (>90), pale yellow shows low confidence (<50).*

*
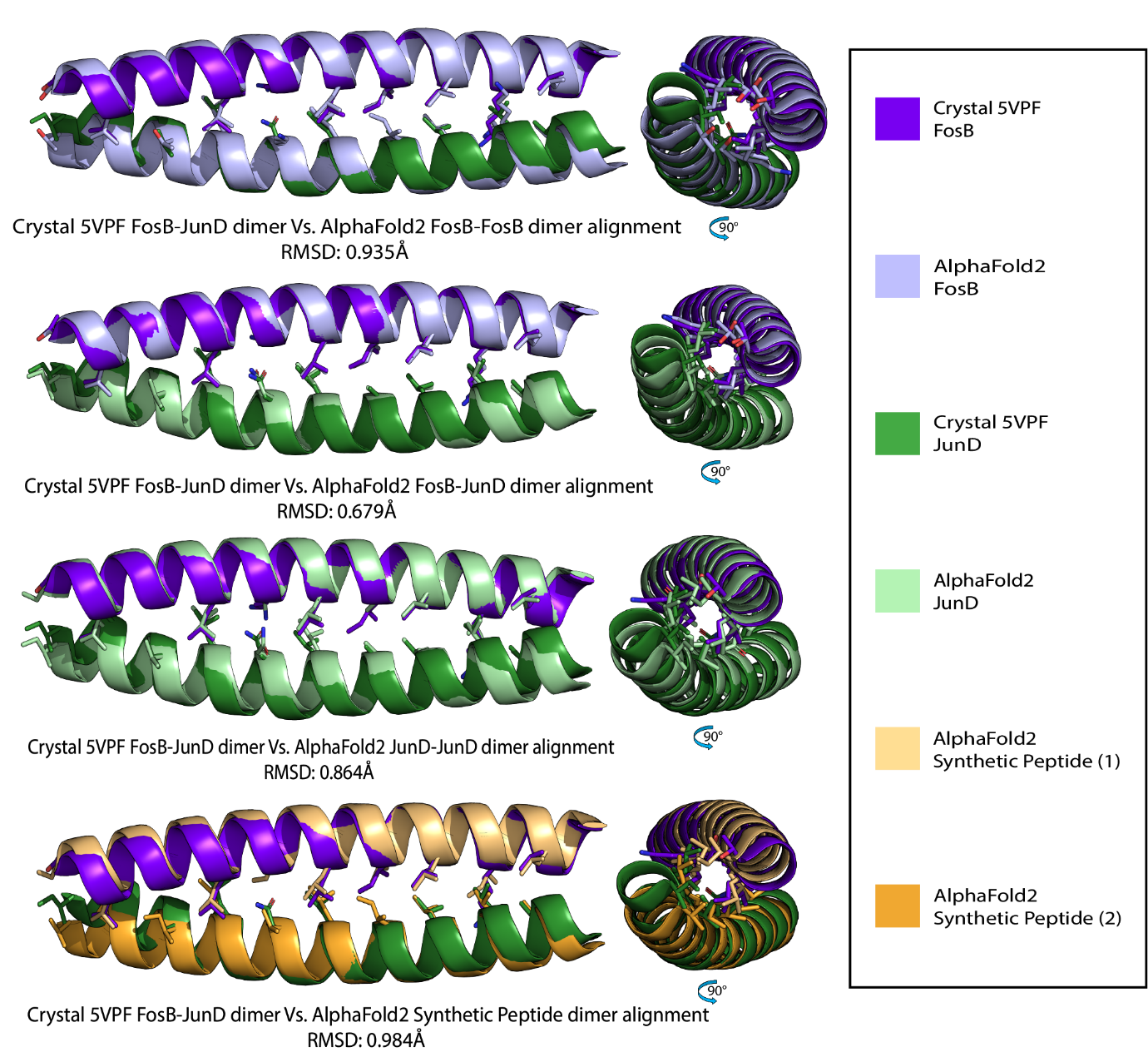
*

*Supplementary Figure 2: Alignments between the crystal structure of the FosB-JunD heterodimer (5VPF) and different AlphaFold2 structures. This demonstrates the close structural alignments between the AlphaFold2 L-zip structures and the experimental L-zip structure.*

*
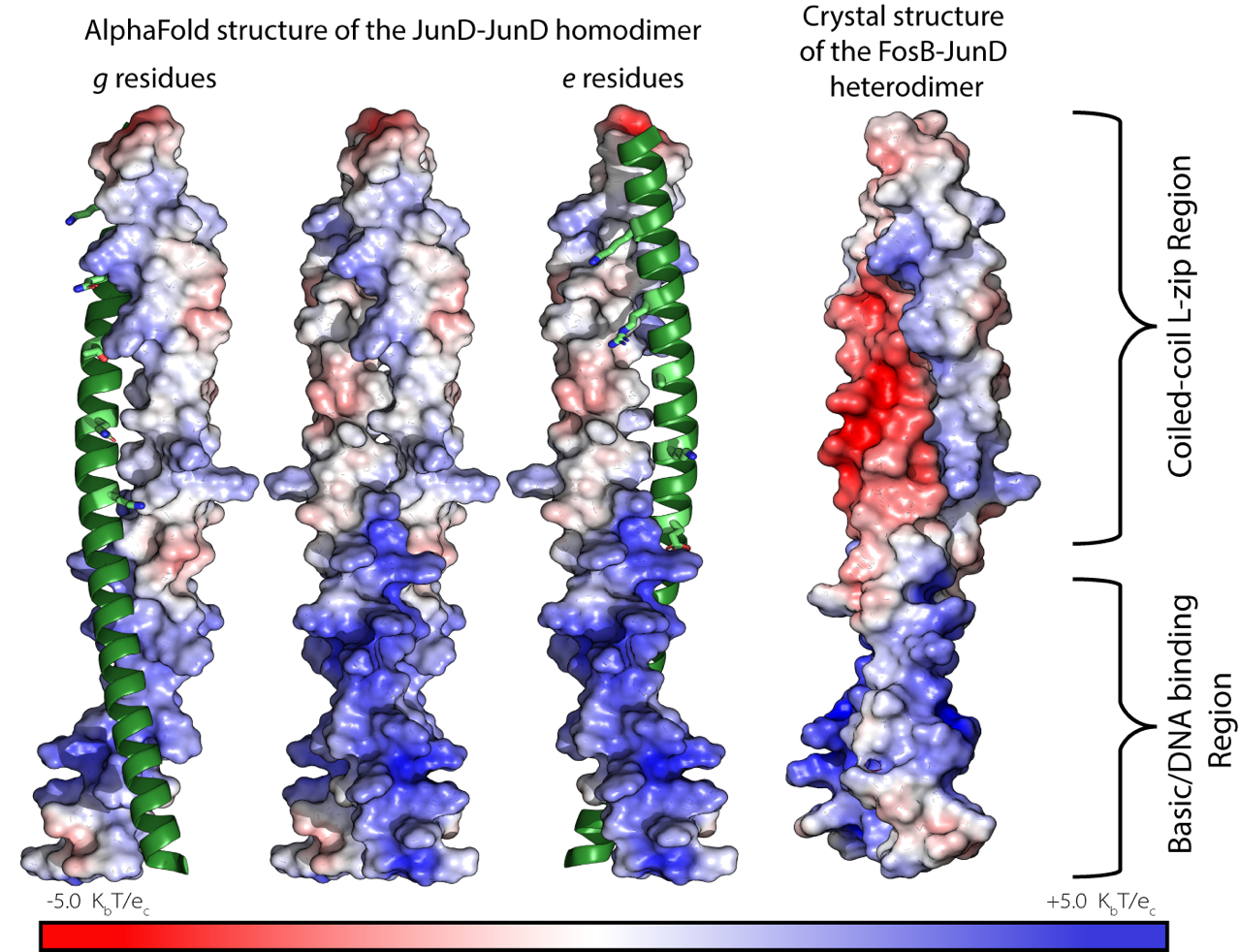
Supplementary Figure 3: Electrostatic surface potential of the structured coiled-coil region of the AlphaFold2 JunD homodimer and the crystal structure FosB-JunD heterodimer. The cartoon representation of JunD is shown in green, and the e or g positioned residues are shown in lime green and as sticks.*


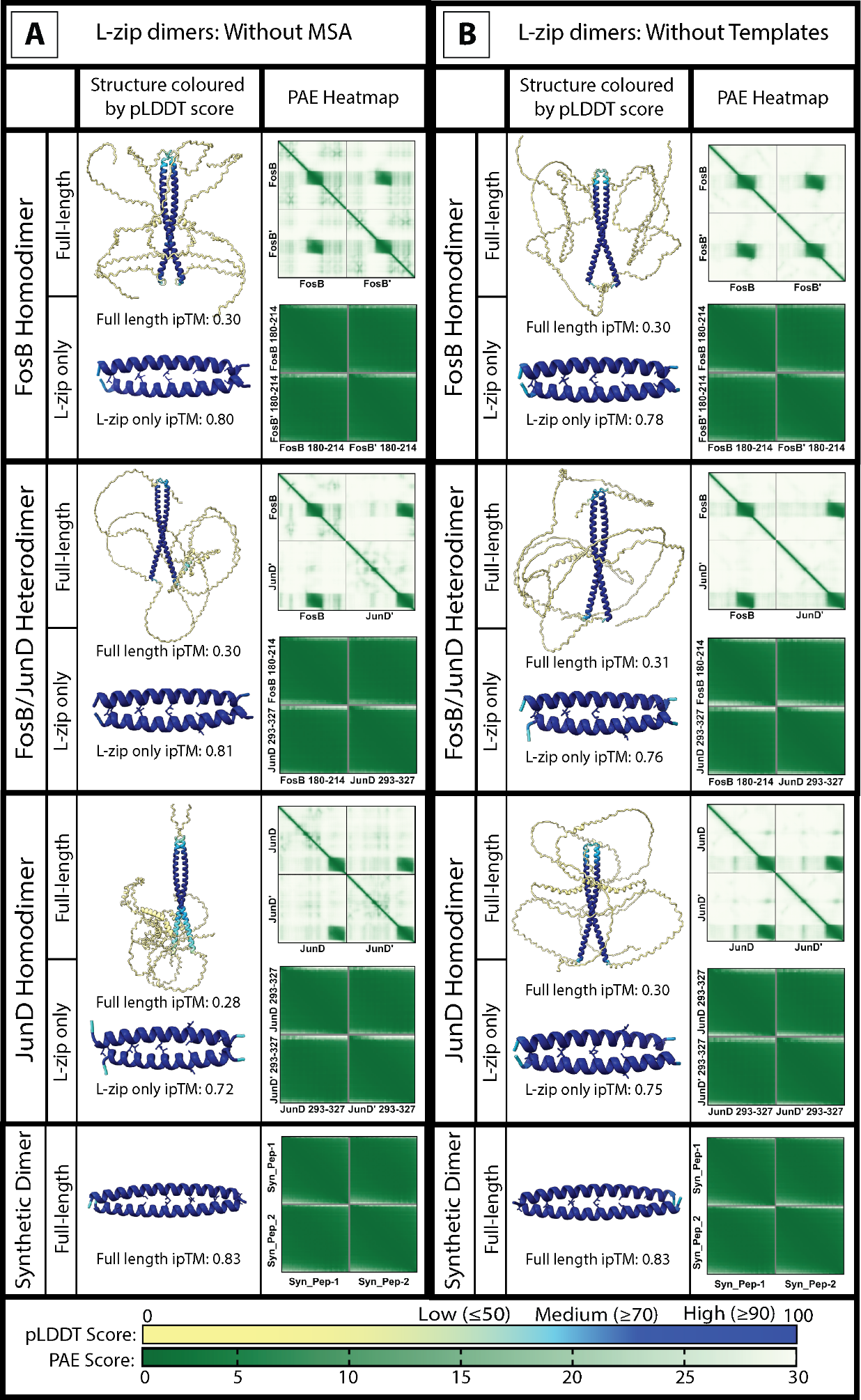


Supplementary Figure 4: An overview of the results of running AlphaFold Multimer without MSA (Panel A) and without structural templates (Panel B). Panel A/B: For the three FosB/JunD dimers, a predicted structure for the full length proteins and an L-zip region only (FosB: T180-V214 and JunD: I293-K327), is shown coloured by pLDDT score. For the L-zip only predicted structures, Leu residues are shown as sticks. A full length predicted structure of our synthetic peptide pair is also displayed, coloured by pLDDT score and the Leu residues are shown as sticks. The corresponding PAE scores are shown as a heat map next to each predicted structure. An ipTM score is shown for each predicted structure.


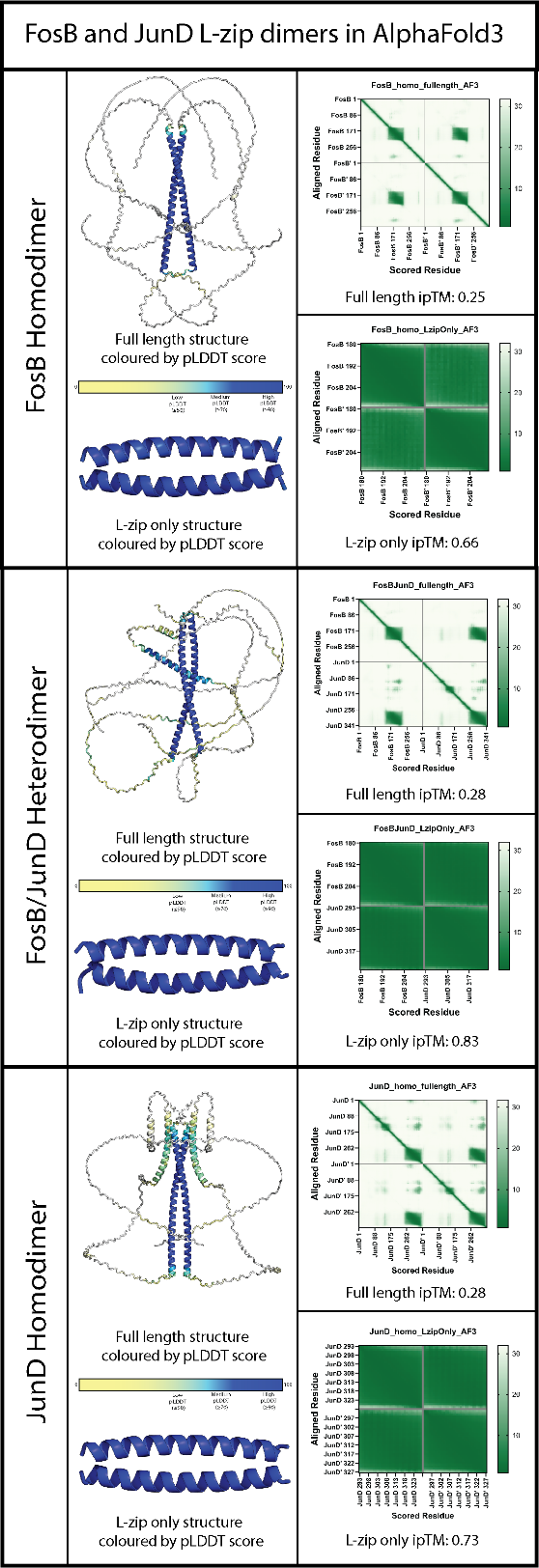


*Supplementary Figure 5: An overview of the results of modelling the three possible FosB/JunD dimers in AlphaFold3 (Google AlphaFold3 server). The predicted structure for the full length proteins and an L-zip region only (FosB: T180-V214 and JunD: I293-K327), is shown coloured by pLDDT score. The corresponding PAE scores are shown as a heat map next to each predicted structure. An ipTM score is shown for each predicted structure.*

*
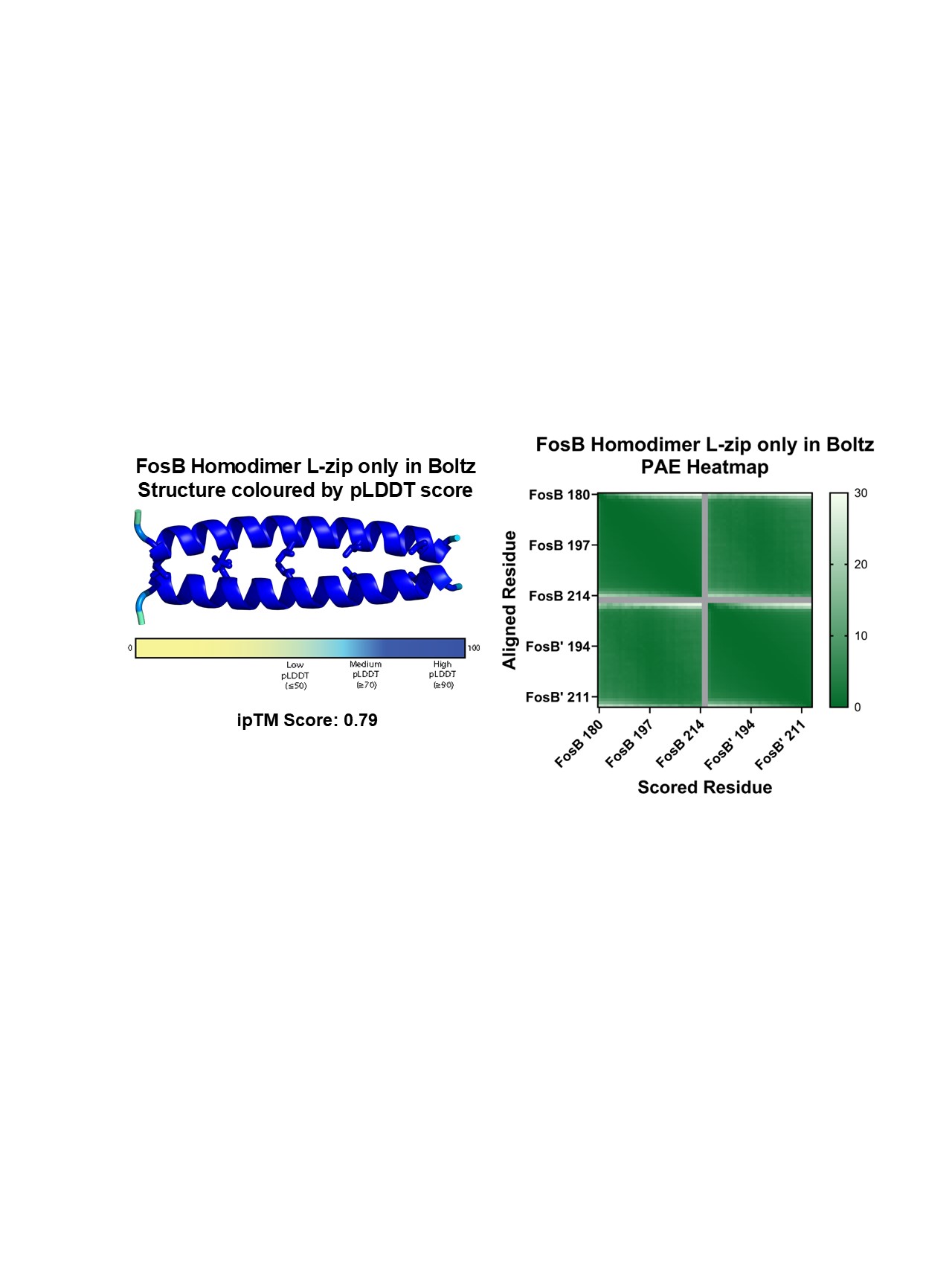
* *Supplementary Figure 6: The minimal L-zip only region of the FosB homodimer structure as predicted by Boltz-1. A predicted structure, PAE scores displayed as a heat map and ipTM score are displayed. The dimerisation data from Newman and Keating, (2003) included a FosB homodimer. In our Boltz predictions, the FosB homodimer did not form an L-zip, contrary to our previous AlphaFold2 predictions (Figure 2A, 6, Supplementary Figure 5). The FosB fragment used by Newman and Keating, (2003) is longer than our 35-residue long L-zip-only fragment at 68 residues. When we used Boltz to predict the structure of our minimal FosB 35-residue L-zip fragment, Boltz predicted an L-zip with high confidence. This indicates that the inclusion of the unstructured regions at either end of the L-zip affected the quality/accuracy of the structural prediction.*


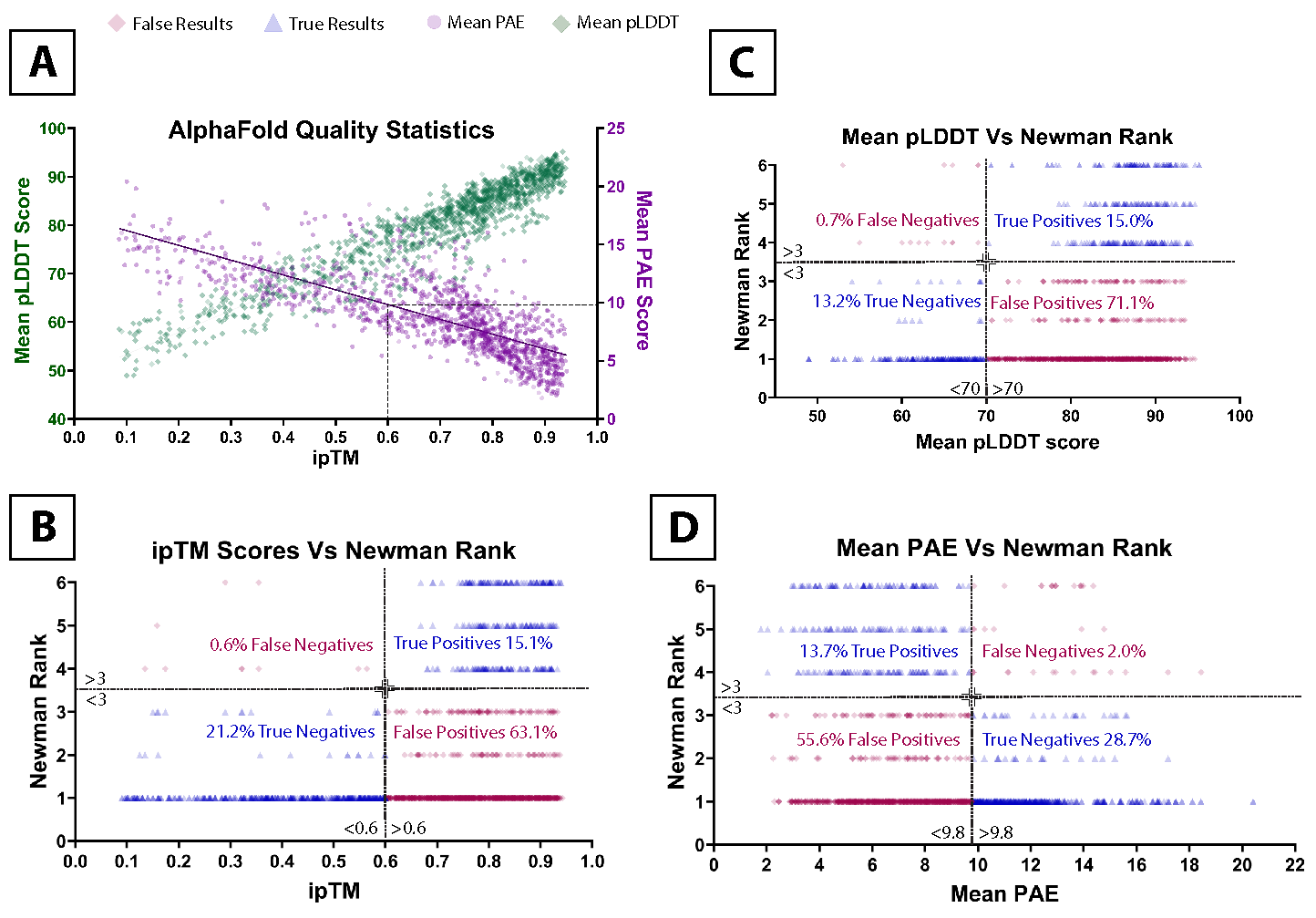


*Supplementary Figure 7: Newman Rank graphs from L-zip dimer predictions from Boltz-1. A: Mean pLDDT scores and Mean PAE scores plotted against ipTM scores. Dashed lines show the intersect of ipTM 0.6 and the Mean PAE score trendline. B, C and D: Scatter graphs of the Newman Rank of L-zip dimers against three different confidence metrics, Mean PAE, ipTM scores and Mean pLDDT scores. True results are shown as blue triangles and are defined as dimers where the Newman rank agrees with the confidence metrics. False results are shown as red diamonds, and are defined as dimers where the Newman rank disagrees with the confidence metrics. Throughout: overlapping data points are shown as darker coloured symbols. In accordance with published guidance (Jumper et al., 2021; Varadi et al., 2021; Evans et al., 2022), ipTM scores of ≥0.6 were considered positive results, and mean pLDDT scores of ≥70 were also considered positive. There is no published threshold for a positive PAE score (Jumper et al., 2021; Varadi et al., 2021; Evans et al., 2022). For the purposes of this study, we chose a threshold mean PAE score of 9.8 based on the correlation between mean PAE and ipTM within this dataset. (Panel A.) This panel also serves to illustrate the broad agreement between these three confidence metrics.*

Supplementary Table 1:

| Heptad Position | FosB Homodimer Ranked_0 Chain_A | | | FosB Homodimer Ranked_0 Chain_B | | | FosB/JunD Heterodimer Ranked_0 Chain_A (FosB) | | FosB/JunD Heterodimer Ranked_0 Chain_B (JunD) | | JunD Homodimer Ranked_0 Chain_A | | JunD Homodimer Ranked_0 Chain_B | |
| --- | --- | --- | --- | --- | --- | --- | --- | --- | --- | --- | --- | --- | --- | --- |
|  | Residue/  Number | pLDDT score | | Residue/  Number | | pLDDT score | Residue/  Number | pLDDT score | Residue/  Number | pLDDT score | Residue/  Number | pLDDT score | Residue/  Number | pLDDT score |
| *a^1^* | THR/180 | | 96.19 | THR/180 | 96.19 | | THR/180 | 97.88 | ILE/293 | 97.44 | ILE/293 | 97.81 | ILE/293 | 97.81 |
| *b^1^* | ASP/181 | | 96.12 | ASP/181 | 96.25 | | ASP/181 | 98.38 | SER/294 | 97.81 | SER/294 | 98.19 | SER/294 | 98.25 |
| *c^1^* | ARG/182 | | 97.19 | ARG/182 | 97.19 | | ARG/182 | 98.44 | ARG/295 | 98.25 | ARG/295 | 98.31 | ARG/295 | 98.31 |
| *d^1^* | LEU/183 | | 97.5 | LEU/183 | 97.56 | | LEU/183 | 98.56 | LEU/296 | 98.38 | LEU/296 | 98.44 | LEU/296 | 98.38 |
| *e^1^* | GLN/184 | | 96.94 | GLN/184 | 96.88 | | GLN/184 | 98.56 | GLU/297 | 98.38 | GLU/297 | 98.5 | GLU/297 | 98.5 |
| *f^1^* | ALA/185 | | 97.19 | ALA/185 | 97.19 | | ALA/185 | 98.62 | GLU/298 | 98.44 | GLU/298 | 98.31 | GLU/298 | 98.44 |
| *g^1^* | GLU/186 | | 97.31 | GLU/186 | 97.38 | | GLU/186 | 98.69 | LYS/299 | 98.62 | LYS/299 | 98.44 | LYS/299 | 98.38 |
| *a^2^* | THR/187 | | 97.12 | THR/187 | 97.12 | | THR/187 | 98.75 | VAL/300 | 98.75 | VAL/300 | 98.44 | VAL/300 | 98.44 |
| *b^2^* | ASP/188 | | 97.25 | ASP/188 | 97.25 | | ASP/188 | 98.75 | LYS/301 | 98.56 | LYS/301 | 98.19 | LYS/301 | 98.25 |
| *c^2^* | GLN/189 | | 97.88 | GLN/189 | 97.88 | | GLN/189 | 98.81 | THR/302 | 98.69 | THR/302 | 98.44 | THR/302 | 98.44 |
| *d^2^* | LEU/190 | | 98.06 | LEU/190 | 98.06 | | LEU/190 | 98.81 | LEU/303 | 98.75 | LEU/303 | 98.5 | LEU/303 | 98.44 |
| *e^2^* | GLU/191 | | 97.38 | GLU/191 | 97.44 | | GLU/191 | 98.75 | LYS/304 | 98.75 | LYS/304 | 97.81 | LYS/304 | 97.88 |
| *f^2^* | GLU/192 | | 96.75 | GLU/192 | 96.81 | | GLU/192 | 98.75 | SER/305 | 98.62 | SER/305 | 97.75 | SER/305 | 97.88 |
| *g^2^* | GLU/193 | | 97.75 | GLU/193 | 97.81 | | GLU/193 | 98.69 | GLN/306 | 98.62 | GLN/306 | 97.62 | GLN/306 | 97.62 |
| *a^3^* | LYS/194 | | 97.75 | LYS/194 | 97.75 | | LYS/194 | 98.75 | ASN/307 | 98.69 | ASN/307 | 96.88 | ASN/307 | 96.75 |
| *b^3^* | ALA/195 | | 96.69 | ALA/195 | 96.75 | | ALA/195 | 98.62 | THR/308 | 98.69 | THR/308 | 96.75 | THR/308 | 96.88 |
| *c^3^* | GLU/196 | | 97.62 | GLU/196 | 97.62 | | GLU/196 | 98.75 | GLU/309 | 98.69 | GLU/309 | 97 | GLU/309 | 97.12 |
| *d^3^* | LEU/197 | | 98.06 | LEU/197 | 98.06 | | LEU/197 | 98.75 | LEU/310 | 98.69 | LEU/310 | 96.56 | LEU/310 | 96.56 |
| *e^3^* | GLU/198 | | 97.56 | GLU/198 | 97.56 | | GLU/198 | 98.69 | ALA/311 | 98.62 | ALA/311 | 94.81 | ALA/311 | 94.81 |
| *f^3^* | SER/199 | | 97.62 | SER/199 | 97.62 | | SER/199 | 98.56 | SER/312 | 98.62 | SER/312 | 96.06 | SER/312 | 96.25 |
| *g^3^* | GLU/200 | | 97.19 | GLU/200 | 97.19 | | GLU/200 | 98.5 | THR/313 | 98.62 | THR/313 | 95.19 | THR/313 | 95.25 |
| *a^4^* | ILE/201 | | 97.88 | ILE/201 | 97.88 | | ILE/201 | 98.62 | ALA/314 | 98.56 | ALA/314 | 94.62 | ALA/314 | 94.5 |
| *b^4^* | ALA/202 | | 97.12 | ALA/202 | 97.12 | | ALA/202 | 98.5 | SER/315 | 98.5 | SER/315 | 94.38 | SER/315 | 94.62 |
| *c^4^* | GLU/203 | | 96.75 | GLU/203 | 96.75 | | GLU/203 | 98.38 | LEU/316 | 98.56 | LEU/316 | 96.44 | LEU/316 | 96.56 |
| *d^4^* | LEU/204 | | 96.69 | LEU/204 | 96.69 | | LEU/204 | 98.19 | LEU/317 | 98.44 | LEU/317 | 95.38 | LEU/317 | 95.31 |
| *e^4^* | GLN/205 | | 96.19 | GLN/205 | 96.19 | | GLN/205 | 98.38 | ARG/318 | 98.31 | ARG/318 | 96.69 | ARG/318 | 96.69 |
| *f^4^* | LYS/206 | | 95.19 | LYS/206 | 95.19 | | LYS/206 | 97.62 | GLU/319 | 98.25 | GLU/319 | 96.12 | GLU/319 | 96.25 |
| *g^4^* | GLU/207 | | 91.62 | GLU/207 | 91.62 | | GLU/207 | 96.94 | GLN/320 | 98.06 | GLN/320 | 94 | GLN/320 | 94.06 |
| *a^5^* | LYS/208 | | 90.31 | LYS/208 | 90.38 | | LYS/208 | 97.12 | VAL/321 | 97.62 | VAL/321 | 92.94 | VAL/321 | 92.75 |
| *b^5^* | GLU/209 | | 87.5 | GLU/209 | 87.62 | | GLU/209 | 96.25 | ALA/322 | 97.31 | ALA/322 | 91.06 | ALA/322 | 91.12 |
| *c^5^* | ARG/210 | | 88.5 | ARG/210 | 88.62 | | ARG/210 | 94.75 | GLN/323 | 96.94 | GLN/323 | 89.88 | GLN/323 | 90.06 |
| *d^5^* | LEU/211 | | 84.62 | LEU/211 | 84.5 | | LEU/211 | 94.06 | LEU/324 | 95.25 | LEU/324 | 85.31 | LEU/324 | 85.44 |
| *e^5^* | GLU/212 | | 82.94 | GLU/212 | 82.88 | | GLU/212 | 93.56 | LYS/325 | 94.19 | LYS/325 | 84.75 | LYS/325 | 84.56 |
| *f^5^* | PHE/213 | | 84.56 | PHE/213 | 84.56 | | PHE/213 | 93.31 | GLN/326 | 94.06 | GLN/326 | 82.75 | GLN/326 | 82.69 |
| *g^5^* | VAL/214 | | 78.62 | VAL/214 | 78.44 | | VAL/214 | 90.81 | LYS/327 | 91.81 | LYS/327 | 78.62 | LYS/327 | 78.75 |

*Table showing the pLDDT scores for the individual residues in the L-zip regions (T180-V214 for FosB and I293-K327 JunD) of the full length AlphaFold Multimer predictions for the three possible FosB and JunD dimers. These pLDDT scores were extracted from the JSON.file for each dimer, and were averaged to generate the mean pLDDT scores quoted in the body of the text.*

Supplementary material: .zip file of all the PDB files for the synthetic peptide pairs structures produced in AlphaFold-Multimer.

File name: Synthetic Leucine zipper construct PDB files.zip

Supplementary material: .zip file of all the cif. files for the human L-zip dimers as named by Newman and Keating, (2003).

File name: leucine_zippers_Boltz_predictions.zip

Supplementary material: Excel file containing the confidence metrics for the predicted structures of the human L-zip dimers as named by Newman and Keating, (2003).

File name: L-zip dimer pairs AlphaFold confidence metrics.xlsx

Supplementary material: Excel file containing the Newman Rank and basic structural characterisation for the human L-zip dimers as named by Newman and Keating, (2003).

File name: L-zip dimer pairs Newman Ranks and stuctures.xlsx
